# Protein phosphatase 2A activators reverse age‐related behavioral changes by targeting neural cell senescence

**DOI:** 10.1111/acel.13780

**Published:** 2023-01-16

**Authors:** Jun Xing, Kehua Chen, Shuaiyun Gao, Mélanie Pousse, Yilin Ying, Bo Wang, Lianxiang Chen, Cuicui Wang, Lei Wang, Weiguo Hu, Yiming Lu, Eric Gilson, Jing Ye

**Affiliations:** ^1^ Geriatric Department, Geriatric Medical Center, Shanghai Ruijin Hospital Shanghai Jiao Tong University School of Medicine Shanghai China; ^2^ International Laboratory in Cancer, Aging and Hematology Shanghai Jiao Tong University School of Medicine/Ruijin Hospital/CNRS/Inserm/Côte d'Azur University Shanghai China; ^3^ IRCAN Côte d'Azur University, CNRS, Inserm Nice France; ^4^ Department of Genetics CHU Nice France

**Keywords:** Age‐related cognitive decline, DNA damage response neural senescence, PPP2R2C, protein phosphatase 2A, senolytics, senotherapy

## Abstract

The contribution of cellular senescence to the behavioral changes observed in the elderly remains elusive. Here, we observed that aging is associated with a decline in protein phosphatase 2A (PP2A) activity in the brains of zebrafish and mice. Moreover, drugs activating PP2A reversed age‐related behavioral changes. We developed a transgenic zebrafish model to decrease PP2A activity in the brain through knockout of the *ppp2r2c* gene encoding a regulatory subunit of PP2A. Mutant fish exhibited the behavioral phenotype observed in old animals and premature accumulation of neural cells positive for markers of cellular senescence, including senescence‐associated β‐galactosidase, elevated levels *cdkn2a/b*, *cdkn1a*, senescence‐associated secretory phenotype gene expression, and an increased level of DNA damage signaling. The behavioral and cell senescence phenotypes were reversed in mutant fish through treatment with the senolytic ABT263 or diverse PP2A activators as well as through *cdkn1a* or *tp53* gene ablation. Senomorphic function of PP2A activators was demonstrated in mouse primary neural cells with downregulated *Ppp2r2c*. We conclude that PP2A reduction leads to neural cell senescence thereby contributing to age‐related behavioral changes and that PP2A activators have senotherapeutic properties against deleterious behavioral effects of brain aging.

## Graphical abstract

During aging, the activity of the Protein Phosphatase 2A (PP2A) declines in the brain of mice and zebrafish, leading to neural cell senescence and aged‐related behavioral changes. This process can be reversed by pharmacological activators of PP2A and senolytics.

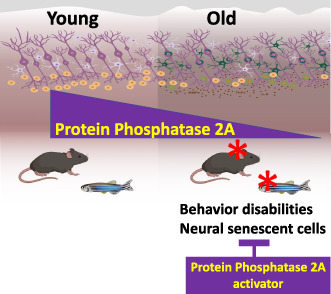

**Abbreviations**
DDR: DNA damage response
MPH: methylphenidate
OT: optic tectum
PP2A: protein phosphatase 2A
ROS: reactive oxygen species
RT‐qPCR: reverse transcription‐quantitative polymerase chain reaction
SASP: senescence‐associated secretory phenotype
WT: wild‐type

## 1 INTRODUCTION

A wealth of recent research indicates that cellular senescence is a basic aging process that greatly contributes to health deterioration and thereby critically impedes healthy aging (Baker et al., 2016; Childs et al., 2017; Song et al., 2020; Tchkonia et al., 2021). Cellular senescence is an essentially permanent arrest of the cell cycle that occurs in response to numerous stressors. This process is accompanied by a permanent activation of the DNA damage response (DDR) and widespread changes in chromatin structure, metabolism, and gene expression, including a senescence‐associated secretory phenotype (SASP) involving the expression and secretion of inflammatory cytokines, growth factors, proteases, and other molecules that can alter tissue microenvironments and cell–cell interactions (Acosta et al., 2013; Correia‐Melo et al., 2016; Wiley et al., 2016). The most prominent senescence‐inducing stimuli are telomere changes, intrinsic and extrinsic sources of genomic and epigenomic damage, activated oncogenes, reactive oxygen species (ROS), and various toxins. An emerging paradigm suggests that senescent cells are major contributors to age‐related illnesses. Indeed, senotherapeutic interventions that counteract cellular senescence either through removal of senescent cells (senolytics) or modification of their phenotype (senomorphic) can restore organ function and slow the aging process (Chang et al., 2016; Di Micco et al., 2021; Gutierrez‐Martinez et al., 2018; Xu et al., 2018; Zhang et al., 2022).

Recent studies have indicated that genes associated with neurological diseases, including Alzheimer's disease, Parkinson's disease, amyotrophic lateral sclerosis, major depressive disorder, bipolar disorder, and schizophrenia, are highly overrepresented among age‐related genes in people with these conditions (Ding et al., 2015; Glorioso et al., 2011). Moreover, evidence indicates that senescent glial cells affect mouse cognition (Ogrodnik et al., 2019) and Alzheimer's disease (Zhang et al., 2019). These observations, together with reports that various types of neuropsychiatric disorders are associated with increased risks of age‐related diseases (Goldstein et al., 2009), mortality (Diniz et al., 2014), and shortened telomeres (Kiecolt‐Glaser & Wilson, 2016), suggest that cellular senescence and premature aging may be important etiologies of brain disorders. In addition to age‐related brain disorders, increasing age is associated with impaired of cognitive abilities (Liu et al., 2015), which affect the quality of life, even for healthy people (Salinas‐Rodríguez et al., 2022). Very little is known about the mechanisms leading to neural cell senescence, age‐related cognitive disabilities, and neurodegenerative diseases.

A candidate protein to connect senescence and age‐related cognitive disabilities is the Ser/Thr protein phosphatase 2A (PP2A), which reverses the phosphorylation of key actors in the DDR, such as γH2AX (Chowdhury et al., 2005; Ferrari et al., 2017). The brain‐isoform PPP2R2C is downregulated in aging brains of wild‐type (WT) and Alzheimer's transgenic mice (Leong et al., 2020). Moreover, it is associated with various mental disorders in humans (Backx et al., 2010; Jacob et al., 2012; Kimura et al., 2019; Xu et al., 2014) and is upregulated by TRF2, which is a telomere capping protein that prevents replicative senescence (Karlseder et al., 2002; Mendez‐Bermudez et al., 2022). Here, we demonstrate in zebrafish and mice that the level of PP2A is reduced in the aged brain. In zebrafish, this reduction led to a neural cell senescence phenotype responsible for a behavioral phenotype characterized by anxiety and hyperactivity. These results pave the way for the use of PP2A activators and senescence modulators to prevent age‐related cognitive disabilities.

## 2 RESULTS

### 2.1 PP2A activity declines in the aging brains of zebrafish and mice

**FIGURE 1:**
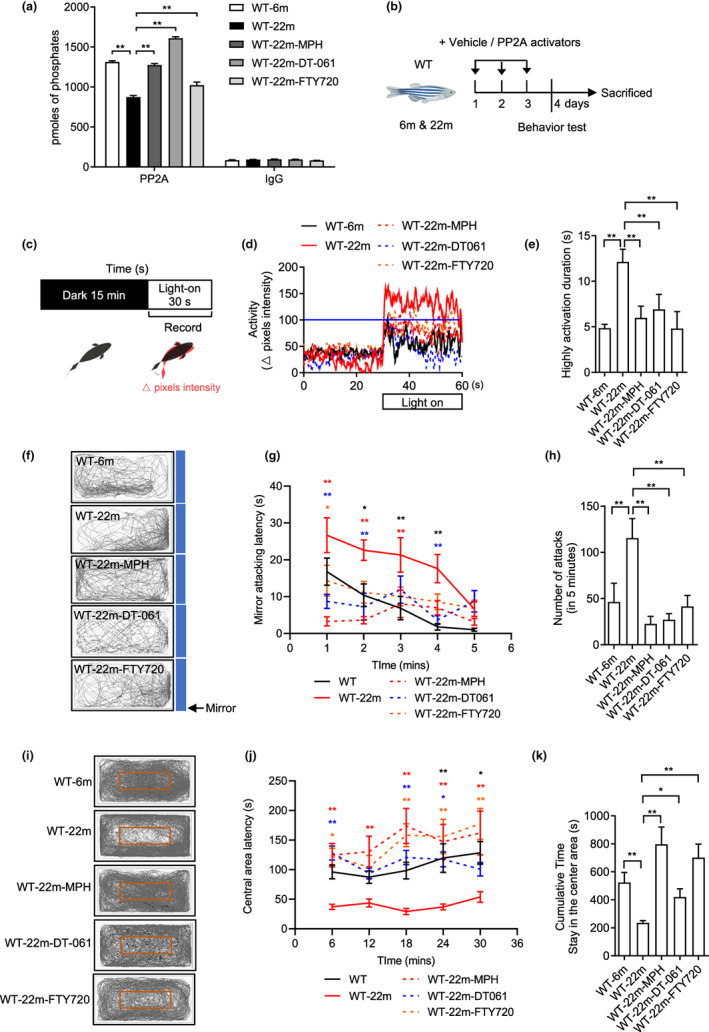
PP2A activators reverse the behavioral changes of old fish. (a) PP2A phosphatase assay after the treatment of PP2A activators in old fish (*n* = 3 independent biological samples, each group contained two brains; one‐way ANOVA). The IgG assays correspond to controls of the PP2A immunoprecipitation. (b) Experimental design of behavioral tests with 6‐month‐old (adult) and 22‐month‐old (old) WT with or without PP2A activators treatment for 3 days. (c) Experimental scheme for the light–dark transition assay. Parameters recorded and analyzed in light–dark transition assay. (d) Representative diagram of locomotion activity during the 30‐s lights‐on period and 30 s before lights‐on. (e) Quantification of the highly activation state (see Section 4) duration during the 30‐s lights‐on period (*n* = 8 for 6 m, *n* = 15 for 22 m, *n* = 11 for 22m‐MPH, *n* = 9 for 22m‐DT‐061 and 22m‐FTY720; one‐way ANOVA). (f) Representative movement tracks (gray lines) in the mirror attack assay of adult fish during the 5‐min trial. Blue boxes show the position of the mirror. (g) Mirror attacking latency at indicated time point in 5‐min trail. (Two‐way ANOVA, statistic difference is shown as WT‐6m vs. WT‐22m (black *), WT‐22m vs. WT‐22m‐MPH (red *), WT‐22m vs. WT‐22m‐DT‐061 (blue *), WT‐22m vs. WT‐22m‐FTY720 (orange *)). (h) Quantification of the number of mirror attacks in 5‐min intervals (*n* = 14 for 6 m, *n* = 15 for 22 m, *n* = 12 for 22m‐MPH, *n* = 9 for 22m‐DT‐061 and 22m‐FTY720; one‐way ANOVA). (i) Representative movement tracks (gray lines) during the 30‐min trial in the open field test. The red box shows the central area of the tank. (j) Central area latency at indicated time point in 30‐min trail. (Two‐way ANOVA, statistic difference are shown as WT‐6m vs. WT‐22m (black *), WT‐22m vs. WT‐22m‐MPH (red *), WT‐22m vs. WT‐22m‐DT‐061 (blue *), WT‐22m vs. WT‐22m‐FTY720 (orange *)). (k) Cumulative time that the adult fish stayed within the central area (*n* = 16 for 6 m, *n* = 8 for 22 m, *n* = 10 for 22m‐MPH, *n* = 9 for 22m‐DT‐061 and 22m‐FTY720; one‐way ANOVA). Data are means ± SEM. *p < 0.05, **p < 0.01

To assess the role of PP2A activity in age‐related brain changes, we first assayed for PP2A phosphatase activity through immunoprecipitation of the PP2A complex (Frohner et al., 2020) from the brains of young and old zebrafish and mice. We observed decreased PP2A activity in both old zebrafish (22 months) and mice (14 months), as compared to young animals (Figures 1a and S1a). Then, we investigated whether this decline can be reversed using three types of pharmacological activators of PP2A: DT‐061 (Leonard et al., 2020), FTY720 (Vicente et al., 2020), and methylphenidate (MPH), a psychostimulant drug with PP2A activator properties (Schmitz et al., 2018). Treatment of aged fish for 3 days with DT‐061(5 mg/kg), FTY720 (5 mg/kg), or MPH (MPH 1.08 mg/kg), respectively, and of aged mice with MPH for 10 days at a dose of 12.3 mg/kg is sufficient to increase the level of PP2A activity as measured via immunoprecipitation with antibodies directly against the PP2A catalytic subunit (Figures 1a and S1a). This PP2A activity augmentation is not accompanied by an increase in the level of the PP2A catalytic subunit (Frohner et al., 2020; Figure S1b), in agreement with a mode of action of the drugs based on the stabilization of the holoenzyme in an active state (Leonard et al., 2020).

### 2.2 Pharmacological activation of PP2A in old zebrafish and mice reverses age‐related behavioral changes

Next, we explored whether the age‐related decline of PP2A is responsible for the behavioral phenotypes observed in aged animals. Compared to young zebrafish, old animals (22 months) exhibited a behavioral phenotype involving increased locomotor activity upon the transition between light and dark (Figure 1c–e), reflecting an abnormal hyperactivity or startling in response to an environmental change, impulsive and aggressive behavior as revealed by continued attacks on a mirror (Huang et al., 2015; Liu & Liu, 2020; Figure 1f–h) and reduced exploratory behavior in an open field test (Roybal et al., 2007; Figure 1i–k), which can be interpreted as an anxiogenic behavior (Egan et al., 2009). Swimming speed of 22‐month‐old fish is not differ from that of young fish (Figure S1c). Notably, in addition to the total time spent in the center of the tank being lower, the longest duration in the center (measured every 6 min) was greatly reduced (Figure 1j). Treatment of the aged fish with DT‐061 or MPH or FTY720 for 3 days was sufficient to reverse their behavioral phenotypes (Figure 1d–k), indicating that the age‐related decline in PP2A activity is responsible for the behavior changes.

Mice at 14 months of age exhibited greater anxiety (Figure S1e), as well as cognitive and learning impairment (Figure S1f,g), relative to WT mice at 3 months, in agreement with previous research (Belblidia et al., 2018; Shoji et al., 2016). Similar to aged zebrafish, treating aged mice with MPH ameliorated the age‐related anxiety phenotype, as well as cognitive and learning deficits, relative to young mice in the light/dark transition and Morris tests (Figure S1d–g).

### 2.3 *ppp2r2c* mutant zebrafish exhibit a behavioral deficit phenotype similar to aged fish

The results presented above encouraged us to develop a transgenic fish model with reduced PP2A activity in the brain. We first confirmed that the *ppp2r2c* gene encoding a brain‐specific isoform of the regulatory subunits of PP2A (Fagerberg et al., 2014) is highly expressed in the zebrafish brain (Figure S2a). Then, we generated two *ppp2r2c* mutant zebrafish lines (*ppp2r2c* ^*m1/m1*^ and *ppp2r2c* ^*m2/m2*^) through the introduction of two different frameshift mutations within exon 9. These mutations led to a reduction of the mutant mRNA level, likely driven by nonsense‐mediated mRNA decay due to the presence of a premature stop codon (Figure S2b,c). The predicted truncated ppp2r2c proteins expressed by the two mutant genes lack the three terminal WD40 repeats. Such a truncation prevents the folding of the protein into a typical circular solenoid WD40 domain (Smith et al., 1999) and is therefore expected to produce a nonfunctional protein. In accordance with the loss of functional *ppp2r2c* and the brain‐specific expression of *ppp2r2c*, the PP2A activity was reduced by roughly 30% in the brain but not in other organs (Figure S2d). Notably, the expression of the catalytic subunit in the phosphatase assay was similar between the WT and mutant brain extracts (Figure S2e), indicating that the reduced level of the regulatory subunit ppp2r2c affects the specific activity of the complexes containing the PP2A catalytic subunit.

**FIGURE 2:**
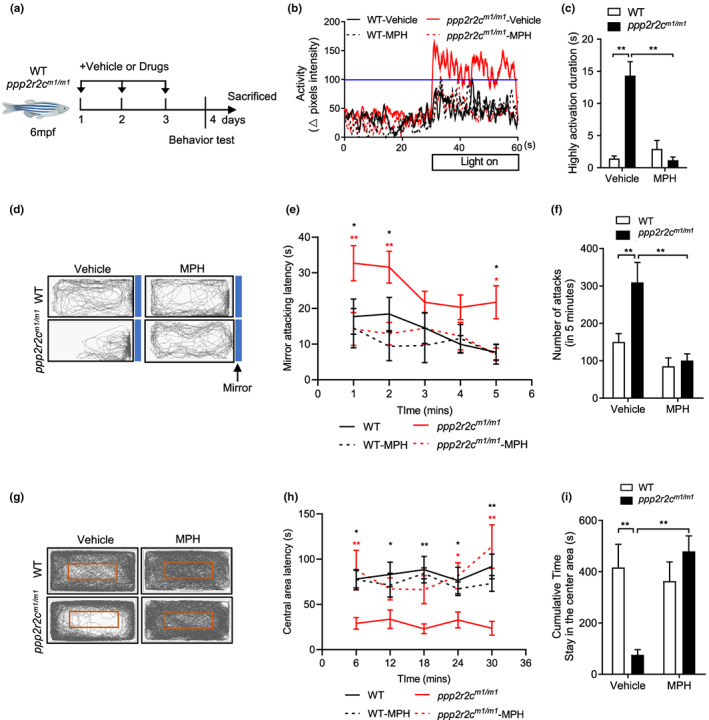
*ppp2r2c*‐compromised fish exhibit abnormal behaviors that are reversed by MPH. (a) Experimental design. Behavioral tests in WT and *ppp2r2c* ^*m1/m1*^ (6 months old) with or without MPH treatment for 3 days. Parameters recorded and analyzed in light–dark transition assay. (b) Representative diagram of locomotion activity of adult fish during the 30‐s lights‐on period and 30 s before lights‐on. (c) Quantification of the highly active state duration during the 30‐s lights‐on period (see Section 4; *n* = 9 for every group). (d) Representative movement tracks (gray lines) in the mirror attack assay of WT and *ppp2r2c* ^*m1/m1*^ (6 months old), with or without MPH treatment for 3 days, during the 5‐min trial. Blue boxes show the position of the mirror. (e) Mirror attacking latency at indicated time point in 5‐min trail. (Statistic difference are shown as WT vs. *ppp2r2c* ^*m1/m1*^ (black *), *ppp2r2c* ^*m1/m1*^ vs. *ppp2r2c* ^*m1/m1*^‐MPH (red *)). (f) Quantification of the number of mirror attacks in the 5‐min intervals (*n* = 9 for WT‐vehicle, WT‐MPH, and *ppp2r2c* ^*m1/m1*^‐vehicle; *n* = 8 for *ppp2r2c* ^*m1/m1*^‐MPH). (g) Representative movement tracks (gray lines) of WT and *ppp2r2c* ^*m1/m1*^ (6 months old), with or without MPH treatment for 3 days, during the 30‐min trial in the open field test. The red square shows the central area of the tank. (h) Central area latency at indicated time point in 30‐min trail. (Statistic difference is shown as WT vs. *ppp2r2c* ^*m1/m1*^ (black *), *ppp2r2c* ^*m1/m1*^ vs. *ppp2r2c* ^*m1/m1*^‐MPH (red *)). (i) Cumulative time that adult fish stayed within the central area (*n* = 7 for *ppp2r2c* ^*m1/m1*^‐vehicle; *n* = 8 for WT‐vehicle and WT‐MPH; *n* = 9 for *ppp2r2c* ^*m1/m1*^‐MPH). Data are means ± SEM. *p < 0.05, **p < 0.01; two‐way ANOVA

Homozygous *ppp2r2c* ^*m1/m1*^ and *ppp2r2c* ^*m2/m2*^ fish had a reduced lifespan (Figure S2f). Remarkably, the adult *ppp2r2c* ^*m1/m1*^ and *ppp2r2c* ^*m2/m2*^ fish exhibited a behavioral deficit phenotype similar to old WT fish, including hyperactivity or a startle response, reflected in an increase in locomotor activity in the light–dark transition assay (Figures 2a–c and S3a,b, see Section 4), which cannot be explained by a change in swimming speed (Figure S3c), continuation of impulsive or aggressive behavior as indicated by a mirror attack assay (Figures 2a,d–f and S3d–f), and anxiety since *ppp2r2c* ^*m1/m1*^ and *ppp2r2c* ^*m2/m2*^ fish were markedly less exploratory than WT fish in an open field test (Figures 2a,g‐i and S3g–i), although the total swimming distance was the same (Figure S3j). These behavioral changes were not accompanied by sleep problems or social withdrawal in *ppp2r2c* ^*m1/m1*^ fish (Figure S3k–m). As the two mutant fish lines exhibited similar behavioral phenotypes, we used *ppp2r2c* ^*m1/m1*^ for further experimentation.

Administration of three types of PP2A pharmacological activators (MPH, DT‐061, and FTY720) to *ppp2r2c* ^*m1/m1*^ fish over 3 days restored PP2A activity (Figure S4a) and reversed their behavioral phenotype (Figures 2a‐i and S4b–g). That these PP2A activators can enhance PP2A enzyme activity in zebrafish lacking the regulatory subunit *ppp2r2c* might be explained by the stabilization of holoenzymes containing other regulatory subunits than the one encoded by the *ppp2r2c* gene (Leonard et al., 2020). Treatment with both MPH and DT‐061 did not show an additive effect in the behavioral tests (Figure S4b–g), further supporting the conclusion that MPH and DT‐061 can reverse behavioral changes through their shared capacity to activate PP2A.

In summary, two different loss‐of‐function mutations in *ppp2r2c* in zebrafish led to the phenotype of aged WT zebrafish, which could be reversed by PP2A activators.

### 2.4 *ppp2r2c* mutations exhibit transcriptional signatures of oxidative stress and uncontrolled replication

To study the mechanisms through which *ppp2r2c* mutation leads to the observed behavioral deficit phenotype, we analyzed the quantities of 23 classical neurotransmitters in the brain of 6‐month‐old *ppp2r2c* ^*m1/m1*^ and WT fish using liquid chromatograph mass spectrometer. Among the detected peaks, the only significant change was an increase in the level of L‐tyrosine in mutant fish and no alterations in the levels of catecholamines, including dopamine, were observed (Figure S5).

**FIGURE 3:**
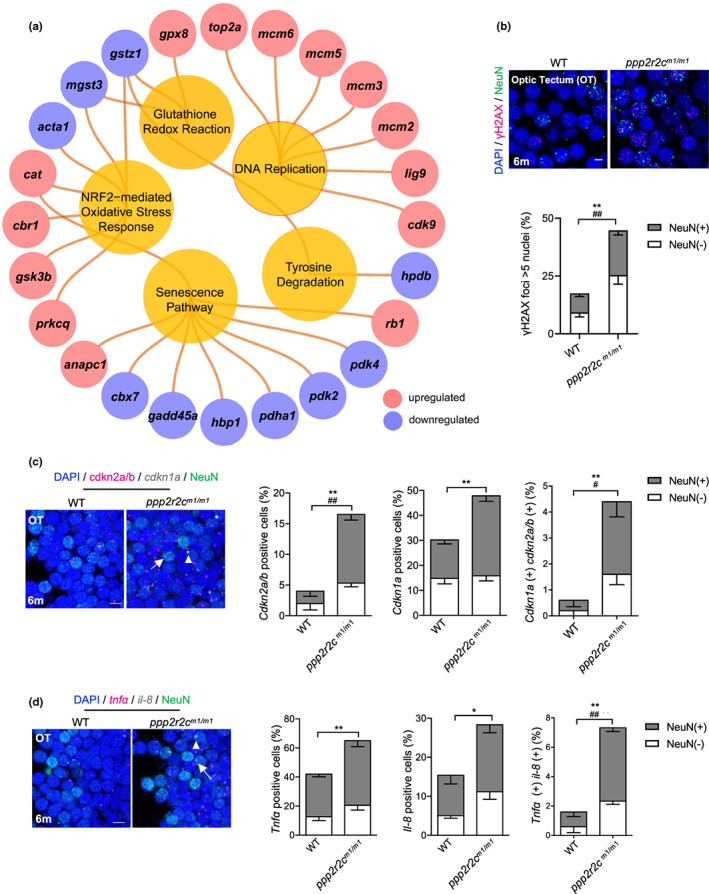
*ppp2r2c*‐compromised fish exhibit an increased rate of neural cells with senescence markers. (a) Ingenuity pathway analysis of *ppp2r2c* ^*m1/m1*^ versus WT brains. Inner circles show selected pathways enriched among the DEGs. Outer circles show upregulated (red) and downregulated (blue) DEGs in *ppp2r2c* ^*m1/m1*^ compared with WT brains (*n* = 3 for every group) (Fisher's exact test, *p* value <0.0001 for DNA replication; *p* value <0.01 for the rest pathway). (b) Representative images of confocal section of immunofluorescence showing NeuN (green) co‐staining with γH2AX (magenta) in the OT of WT and *ppp2r2c* ^*m1/m1*^ at 6 months old (scale bars, 5 μm). Quantification of γH2AX‐positive (positive values indicate at least five γH2AX foci) neuronal (NeuN+) and non‐neuronal (NeuN−) cells (*n* = 6 for each group and over 100 nuclei were analyzed per fish; * represents statistical difference in NeuN+ group, ^*#*^ represents statistical difference in NeuN− group). (c) Representative images of confocal section of NeuN (green) co‐staining with *cdkn2a/b* (magenta) and *cdkn1a* (gray) mRNA by RNA‐Scope in the OT of WT and *ppp2r2c* ^*m1/m1*^ at 6 months old (scale bars, 5 μm). Arrows and triangles point to the *cdkn2a/b* (magenta) *and cdkn1a* (grey) mRNA signal, respectively. Quantification of the percentage of *cdkn2a/b*, *cdkn1a* positive cells, respectively (positive values indicate at least one mRNA signal), and percentage of double positive cells in neuronal (NeuN+) and non‐neuronal (NeuN−) cells (*n* = 5 for each group and over 100 nuclei were analyzed per fish; * represents statistical difference in NeuN+ group, ^*#*^ represents statistical difference in NeuN− group). (d) Representative images of confocal section of NeuN (green) co‐staining with *tnfa* (magenta) and *il‐8* (gray) mRNA by RNA‐Scope in the OT of WT and *ppp2r2c* ^*m1/m1*^ at 6 months old (scale bars, 5 μm). Arrows and triangles point to the *tnfa* and *il‐8* mRNA signals, respectively. Quantification of the percentage of *tnfa*, *il‐8‐*positive cells, respectively (positive values indicate at least one mRNA signal) and percentage of double positive cells in neuronal (NeuN+) and non‐neuronal (NeuN−) cells (*n* = 5 for each group and over 100 nuclei were analyzed per fish; * represents statistical difference in NeuN+ group, ^#^ represents statistical difference in NeuN− group). Data are means ± SEM. *p < 0.05, **p < 0.01, ^#^ *p* < 0.05, ^##^ *p* < 0.01; unpaired two‐sided *t* test.

We next investigated the transcriptomic changes induced by the *ppp2r2c* mutation using RNA sequencing (RNA‐seq) analysis. Comparison of the *ppp2r2c* ^*m1/m1*^ and WT brain transcriptomes revealed differential expression of 473 genes (Table S1; Figure S6a). The differential expression of eight genes was checked using reverse transcription‐quantitative polymerase chain reaction (RT‐qPCR; Figure S6b). Analysis of Gene Ontology terms and Ingenuity pathways indicated marked deregulation of age‐related processes such as cell cycle control, DNA repair, L‐tyrosine catabolism, and antioxidant pathways (Figures 3a and S6c). Transcriptional downregulation of two genes in the L‐tyrosine catabolism pathway might contribute to the increased L‐tyrosine level (Figures 3a and S5). Augmentation of oxidative defense was correlated with increased levels of ROS in the brains of the mutant fish (Figure S6d). The brains of the *ppp2r2c* ^*m1/m1*^ fish also overexpressed genes involved in chromosomal DNA replication, such as subunits of the replicative helicase subunits (MCM), Top2A and ligase 1 (Figures 3a and S6b).

### 2.5 Tectal cells of *ppp2r2c* mutant fish exhibit senescence markers, apoptosis, and signs of replication stress

The transcriptional signatures described above suggested that the *ppp2r2c* leads to genomic instability and aberrant cell cycle control in the brain. Indeed, adult *ppp2r2c* ^*m1/m1*^ (6‐month‐old) brain cells showed increased DNA damage signaling, as revealed by a higher proportion of nuclei harboring at least five γH2AX foci specifically in the optic tectum (OT; Figures 3b and S7a–c). An increase in apoptotic cells was also detected in the OT of 6‐month‐old *ppp2r2c* ^*m1/m1*^ fish (Figure S7d). The specificity in OT localization of these cellular defects is likely to stem from the higher level of *ppp2r2c* mRNA expression in the OT compared to other parts of the brain (Figure S7e,f). These results indicate that *ppp2r2c* plays an important role in protecting tectal cells against unwanted DDR activation and death.

We then explored whether the increased level of γH2X in the OT is accompanied by other markers of cellular senescence. In 6‐month‐old *ppp2r2c* ^*m1/m1*^ fish, we observed an increased number of cells exhibiting senescence‐associated β‐galactosidase (SA‐β‐gal) specifically in the OT (Figure S7g–i) and increased expression of checkpoint and SASP genes as revealed using RNAscope at the single cell level in the OT (Figure 3c,d), as well as by RT‐qPCR and (Figure S7j). In accordance with the brain‐specific expression of *ppp2r2c*, the senescence markers were absent from the heart or kidney cells of age‐matched fish (Figure S7k–n).

In summary, *ppp2r2c* ^*m1/m1*^ fish exhibited several outcomes associated with DDR activation, including apoptosis (based on the terminal deoxynucleotidyl transferase dUTP nick end labeling assay), DNA damage signaling (γH2X, cell‐cycle checkpoint gene expression), and senescence (SA‐β‐gal and SASP gene expression). This was seen both in tectal cells stained for NeuN (NeuN (+)), which marks developing, immature and mature neurons, and in cells that are NeuN (−). These results indicate that multiple types of neural tectal cells are altered upon *ppp2r2c* inhibition.

### 2.6 Pharmacological PP2A activators reduce the senescence markers in *ppp2r2c* mutant brains

We investigated the effects of PP2A activators on the transcriptional and cellular alterations caused by *ppp2r2c* loss. To this end, we used RNAseq to identify genes with dysregulated expression in *ppp2r2c* ^*m1/m1*^ compared to WT fish rescued by MPH treatment. We found 101 differentially expressed genes in the comparisons of *ppp2r2c* ^*m1/m1*^ with WT and *ppp2r2c* ^*m1/m1*^ without and with treatment (Table S2; Figure S8a–d). Notably, in these two situations, the genes were differentially expressed in opposite directions, demonstrating that they are aberrantly expressed due to *PPP2R2C* loss but rescued by MPH treatment. This opposite pattern direction of differential gene expression was confirmed using RT‐qPCR (Figure S8e).

**FIGURE 4:**
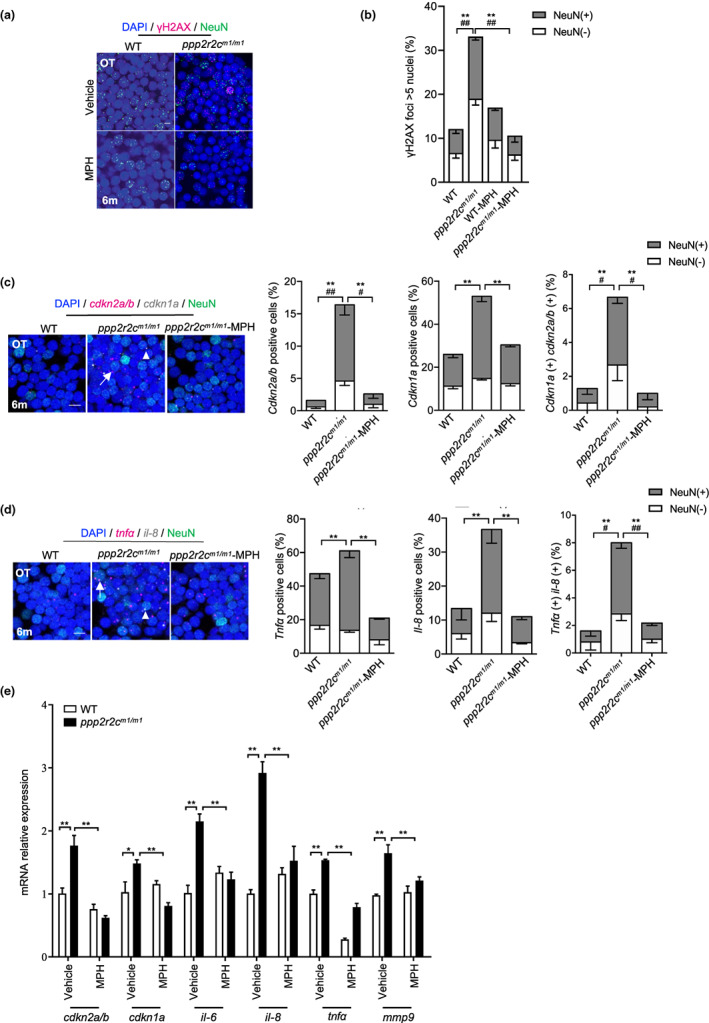
MPH treatment decreases the rate of neural cells with senescence markers in the *ppp2r2c*‐compromised fish. (a) Immunofluorescence detection of NeuN (green) and γH2AX (magenta) in the OT of WT and *ppp2r2c* ^*m1/m1*^ (6‐month‐old) treated with or without MPH for 3 days (scale bars, 5 μm). (b) Quantification shows the percentage of γH2AX positive (positive values indicate at least five γH2AX foci in the nucleus) neuronal (NeuN+) and non‐neuronal (NeuN−) nuclei in (a) (*n* = 6 brains per group and over 100 nuclei were analyzed per fish; * represents statistical difference in NeuN+ group, ^*#*^ represents statistical difference in NeuN− group; one‐way ANOVA). (c) Representative images of confocal section of NeuN (green) co‐staining with *cdkn2a/b* (magenta) and *cdkn1a* (gray) mRNA by RNA‐Scope in the OT of WT and *ppp2r2c* ^*m1/m1*^ treated with or without MPH for 3 days at 6 months old (scale bars, 5 μm). Arrows and triangles point to the *cdkn2a/b* and *cdkn1a* mRNA signals, respectively. Quantification of the percentage of *cdkn2a/b*, *cdkn1a* positive cells, respectively (positive values indicate at least one mRNA signal) and the percentage of double positive cells in neuronal (NeuN+) and non‐neuronal (NeuN−) cells (*n* = 5 for each group and over 100 nuclei were analyzed per fish; * represents statistical difference in NeuN+ group, ^*#*^ represents statistical difference in NeuN− group; one‐way ANOVA). (d) Representative images of confocal section of NeuN (green) co‐staining with *tnfa* (magenta) and *il‐8* (gray) mRNA by RNA‐Scope in the OT of WT and *ppp2r2c* ^*m1/m1*^ treated with or without MPH for 3 days at 6 months old (scale bars, 5 μm). Arrows and triangles point to the *tnfa* and *il‐8* mRNA signals, respectively. Quantification of the percentage of *tnfa*, *il‐8*‐positive cells, respectively (positive values indicate at least one mRNA signal) and percentage of double positive cells in neuronal (NeuN+) and non‐neuronal (NeuN−) cells (*n* = 5 for each group and over 100 nuclei were analyzed per fish; * represents statistical difference in NeuN+ group, ^#^ represents statistical difference in NeuN− group; one‐way ANOVA). (e) Relative mRNA expression levels of *cdkn1a*, *cdkn2a/b*, and key SASP components determined by RT‐qPCR in WT and *ppp2r2c* ^*m1/m1*^ brains with or without MPH treatment (*n* = 3 independent biological samples, every sample pool two brains; two‐way ANOVA). Data are means ± SEM. **p < 0.01, ^#^ *p* < 0.05, ^##^ *p* < 0.01

The first two representative pathways enriched in these genes, which are identical to the pathways altered in mutant fish in comparison with WT controls, were “cell cycle control of chromosomal replication” and “senescence pathway” (Figure S8d). The level of γH2X‐positive cells was reduced in both the neuronal (NeuN (+)) and non‐neuronal (NeuN (−)) tectal cells of 6‐month‐old *ppp2r2c* ^*m1/m1*^ fish treated for 3 days with MPH (Figures 4a,b and S8f). Moreover, MPH mainly decreased the amount of SA‐β‐gal‐positive NeuN (+) cells (Figure S8g) and the expressed levels of checkpoint and SASP genes, as revealed by monitoring with RNAscope (Figure 4c,d) and RT‐qPCR (Figure 4e). No genes whose expression levels were restored by MPH are known targets of MPH in the dopamine pathway. In particular, the *dat1/slc6a3* gene encoding the dopamine transporter was not among the 101 MPH‐restored genes (Table S2). This finding supports the uncoupling of the MPH activity in *ppp2r2c* mutant fish and the dopamine pathway, as suggested by the absence of catecholamine modulation in the mutant fish (Figure S5).

Next, we investigated whether the other PP2A activators also rescued the senescence‐like abnormalities of tectal cells in mutant fish. Indeed, DT‐061 and FTY720 preferentially decreased the DNA damage and SA‐β‐gal index in tectal NeuN (+) cells (Figure S8f,g). Moreover, *ppp2r2c* mutant fish treated with both DT‐061 and MPH had no additional effect on the level of neuronal senescence (Figure S8f,g). These data support the notion that the mode of action of MPH in *ppp2r2c* mutant fish is better explained by its PP2A activator properties than its activity in the dopamine pathway. Based on these results, we conclude that *ppp2r2c* inhibition leads to a senescence‐like phenotype in the OT that can be alleviated by treatment with PP2A activators.

### 2.7 Targeting senescence alleviates behavioral abnormalities in *ppp2r2c* ^*m1/m1*^ fish

The results presented above, namely that PP2A activators alleviate the behavioral abnormalities while also reversing senescence markers suggest that tectal cell senescence is involved in the behavioral deficit phenotype of *ppp2r2c* ^*m1/m1*^ fish. Therefore, we tested whether preventing or reversing senescence is sufficient to restore the behavioral deficits of mutant fish.

**FIGURE 5:**
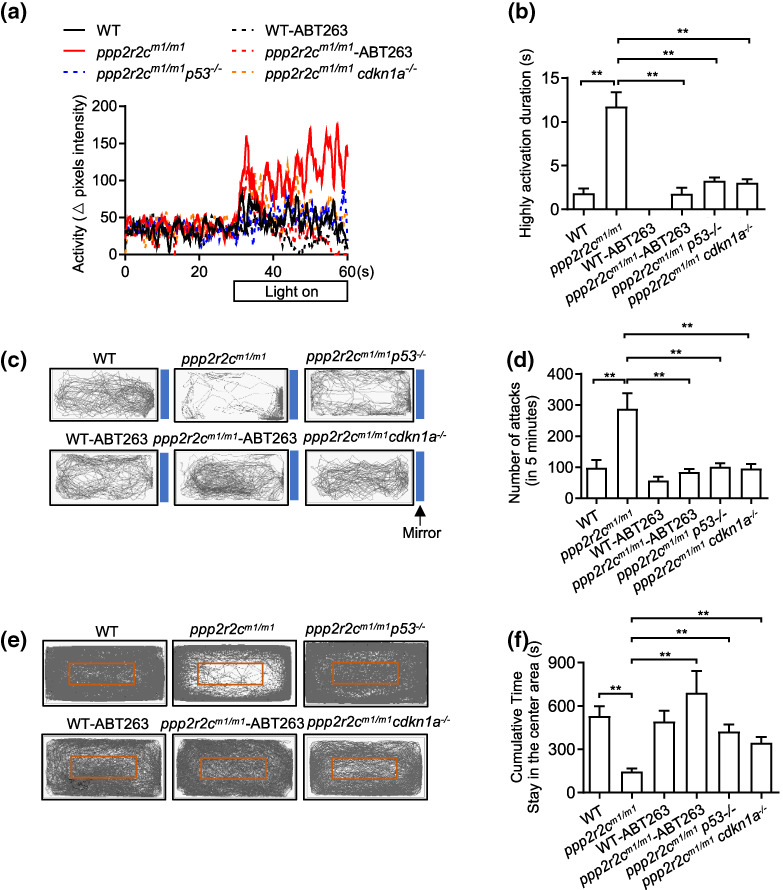
Inhibiting senescence decreases the behavioral disabilities of *ppp2r2c*‐compromised fish. Behavioral tests of WT and *ppp2r2c* ^*m1/m1*^ with or without ABT263 treatment for 3 days, *ppp2r2c* ^*m1/m1*^ *p53* ^*−/−*^ and *ppp2r2c* ^*m1/m1*^ *cdkn1a* ^*−/−*^ at 6 months old. (a) Representative result from locomotion activity upon light–dark transition assay during the 30‐s lights‐on period and 30 s before lights‐on. (b) Quantification of the highly active state duration during the 30‐s lights‐on period (*n* = 11 for *ppp2r2c* ^*m1/m1*^ *p53* ^*−/−*^; *n* = 10 for WT‐vehicle, *ppp2r2c* ^*m1/m1*^‐vehicle and *ppp2r2c* ^*m1/m1*^ *cdkn1a* ^*−/−*^ groups; *n* = 9 for WT‐ABT263; *n* = 8 for *ppp2r2c* ^*m1/m1*^‐ABT263). (c) Representative movement tracks (gray lines) of WT and *ppp2r2c* ^*m1/m1*^ with or without ABT263 treatment for 3 days, *ppp2r2c* ^*m1/m1*^ *p53* ^*−/−*^ and *ppp2r2c* ^*m1/m1*^ *cdkn1a* ^*−/−*^ in the mirror image attack assay. Blue boxes show the position of the mirror. (d) Quantification of the number of mirror attacks in 5‐min intervals (*n* = 11 for *ppp2r2c* ^*m1/m1*^ *p53* ^*−/−*^; *n* = 10 for WT*‐*vehicle, *ppp2r2c* ^*m1/m1*^ *‐*vehiclem *and ppp2r2c* ^*m1/m1*^ *cdkn1a* ^*−/−*^ groups; *n* = 9 for WT‐ABT263 *and ppp2r2c* ^*m1/m1*^ *‐*ABT263). (e) Representative movement tracks (gray lines) of WT and *ppp2r2c* ^*m1/m1*^ with or without ABT263 treatment for 3 days, *ppp2r2c* ^*m1/m1*^ *p53* ^*−/−*^ and *ppp2r2c* ^*m1/m1*^ *cdkn1a* ^*−/−*^ during a 30‐min trial in the open field test. The red box shows the central area of the tank. (f) Quantification of the cumulative time that fish stayed within the central area (*n* = 10 for WT‐vehicle, *ppp2r2c* ^*m1/m1*^ *‐*vehicle, and *ppp2r2c* ^*m1/m1*^ *cdkn1a* ^*−/−*^ groups; *n* = 9 for WT‐ABT263, *ppp2r2c* ^*m1/m1*^ *‐*ABT263, and *ppp2r2c* ^*m1/m1*^ *p53* ^*−/−*^). Data are means ± SEM. **p < 0.01; one‐way ANOVA

Inactivation of two key checkpoint genes (*tp53* and *cdkn1a*) that trigger senescence or treatment with the senolytic drug ABT263, which triggers apoptosis of senescent cells (Wu et al., 2016), relieved the behavioral abnormalities of *ppp2r2c* ^*m1/m1*^ fish (Figures 5a–f and S9a,b). As expected, ABT263 treatment and *tp53* inactivation decreased the number of senescent tectal cells in the *ppp2r2c* ^*m1/m1*^ fish brains (Figure S9c–e). In accordance with tp53 functioning downstream of γH2AX activation, its loss only barely affected the number of γH2X‐positive cells (Figure S9e). The pro‐apoptotic targets of tp53 are likely not responsible for the behavioral phenotype of the *ppp2r2c* ^*m1/m1*^ fish, as the behavioral phenotype is rescued by the ablation of *cdkn1a* (Figure 5a–f), a target of tp53 that does not trigger apoptosis.

### 2.8 PP2A activators reverse senescence markers in the brains of aged fish

**FIGURE 6:**
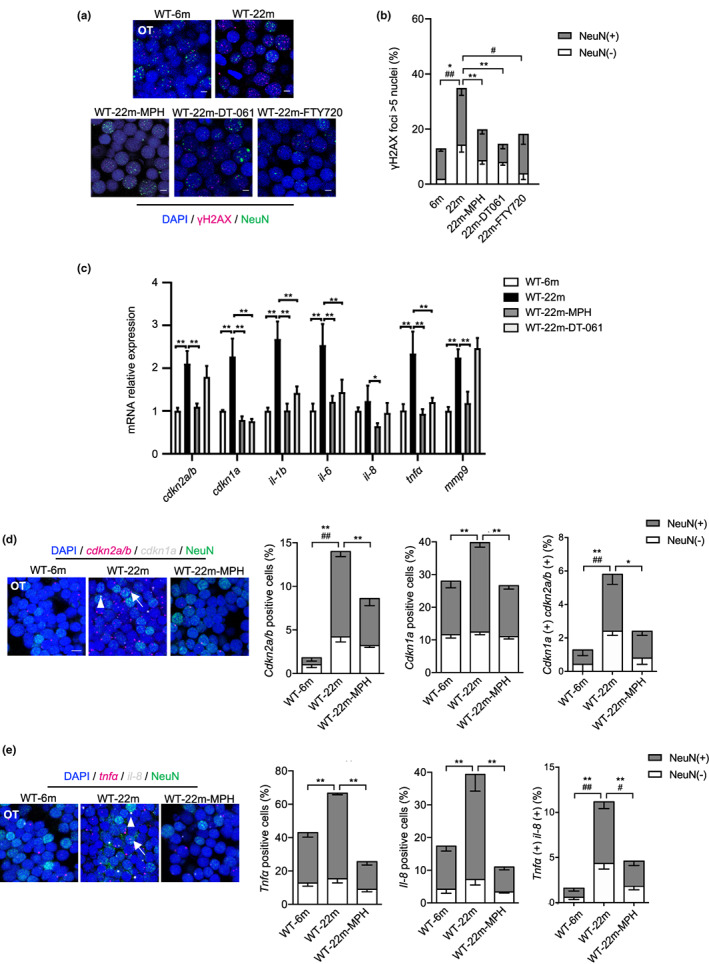
PP2A activators decrease the rate of neural cells with senescence markers in old fish. (a) Representative confocal image of NeuN (green) and γH2AX (magenta) co‐staining in the brain of 22‐month‐old fish treated with or without PP2A activators for 3 days (scale bars, 5 μm). (b) Quantification of γH2AX‐positive nuclei (positive values indicate at least five γH2AX foci) in (a) (*n* = 4 for 6 and 22 m; *n* = 7 for 22m‐MPH, *n* = 3 for 22m‐DT‐061 and 22m‐FTY720, over 100 nuclei were analyzed per fish; * represents statistical difference in NeuN+ group, ^*#*^ represents statistical difference in NeuN− group; one‐way ANOVA). (c) Relative mRNA expression levels of *cdkn1a*, *cdkn2a/b*, and key SASP components determined by RT‐qPCR in 6 and 22 m WT brains with or without MPH or DT‐061 treatment (*n* = 3 independent biological samples, every sample pool two brains; one‐way ANOVA). (d) Representative image of confocal section of NeuN (green) co‐staining with *cdkn2a/b* (magenta) and *cdkn1a* (gray) mRNA by RNA‐Scope in the OT of 6 and 22 m WT treated with or without MPH for 3 days (scale bars, 5 μm). Arrows and triangles point to the *cdkn2a/b* and *cdkn1a* mRNA signals, respectively. Quantification of the percentage of *cdkn2a/b*, *cdkn1a* positive cells, respectively (positive values indicate at least one mRNA signal) and percentage of double positive cells in neuronal (NeuN+) and non‐neuronal (NeuN−) cells (*n* = 5 for each group and over 100 nuclei were analyzed per fish, * represents statistical difference in NeuN+ group, ^#^ represents statistical difference in NeuN− group; one‐way ANOVA). (e) Representative images of confocal section of NeuN (green) co‐staining with *tnfa* (magenta) and *il‐8* (gray) mRNA by RNA‐Scope in the OT of 6 and 22 m WT treated with or without MPH for 3 days (scale bars, 5 μm). Arrows and triangles point to the *tnfa* and *il‐8* mRNA signals, respectively. Quantification of the percentage of *tnfa*, *il‐8‐*positive cells, respectively (positive values indicate at least one mRNA signal) and percentage of double positive cells in neuronal (NeuN+) and non‐neuronal (NeuN−) cells (*n* = 5 for each group and over 100 nuclei were analyzed per fish; * represents statistical difference in NeuN+ group, ^#^ represents statistical difference in NeuN− group; one‐way ANOVA). Data are shown in means ± SEM. *p < 0.05, **p < 0.01, ^#^ *p* < 0.05, ^##^ *p* < 0.01

Next, we asked whether, similar to *ppp2r2c* mutant fish, PP2A activators could reverse neural cell senescence in old animals. We found that 22‐month‐old WT fish exhibit increased levels of γH2X and SA‐β‐gal‐positive cells mostly in the OT (Figures 6a,b and S10a,b), along with elevated level of SASP gene expression (Figure 6c–e) as compared to 6‐month‐old WT fish. When aged WT fish were treated with MPH, DT‐061, and FTY720 for 3 days, the number of γH2X‐positive cells in the OT was markedly reduced (Figure 6a,b). In addition, MPH and DT‐061 treatment of aged fish reduced SASP gene expression (Figure 6c) and SA‐β‐gal (Figure S10a,b). The single cell levels of *cdkn2a/b* ^*p16*^, *cdkn1a*, and cytokine gene transcription were reduced in NeuN (+) cells of aged fish after MPH treatment (Figure 6d,e).

### 2.9 *Ppp2r2c* loss triggers aberrant replication, DDR, and senescence in mouse neural cells

The results described above suggest that reduction of PP2A activity in the brain, resulting from either *ppp2r2c* mutation or natural aging, leads to increased levels of senescence markers in brain cells. Therefore, we investigated the capacity of ppp2r2c loss to trigger senescence in well‐defined mouse neural cell models.

**FIGURE 7:**
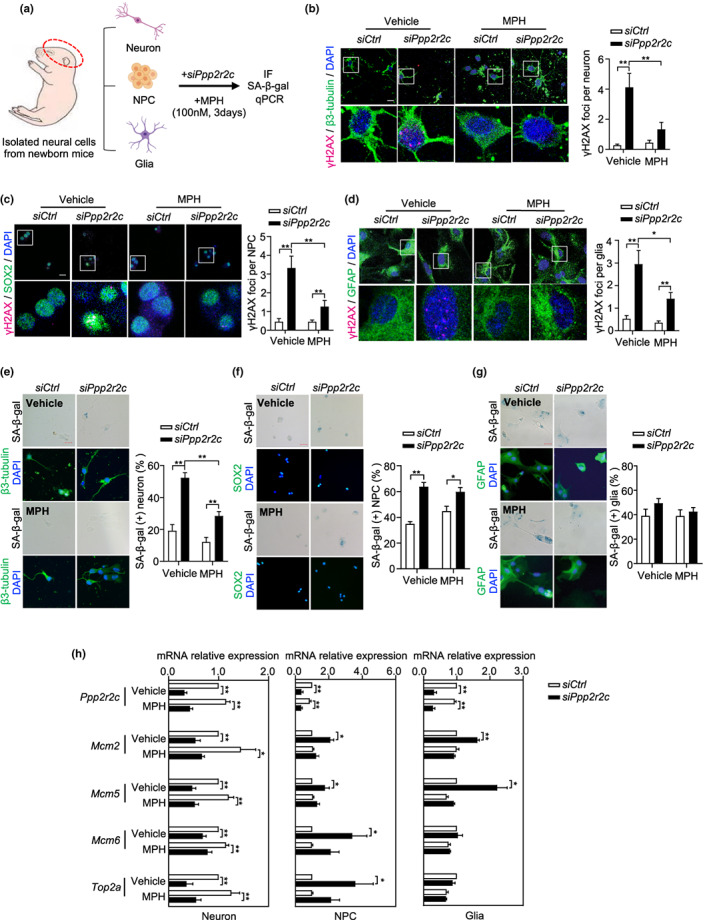
MPH attenuates H2AX phosphorylation induced by *ppp2r2c* knockdown in mice primary neural cells. (a) Experimental design for MPH treatment of *ppp2r2c* knockdown in isolated primary neural cells from newborn mice. (b–d) Representative confocal images and relevant quantification of γH2AX foci (magenta) in primary neurons (stained with β‐tubulin, green), neural progenitor cells (NPCs, stained with SOX2, green), and glial cells (stained with GFAP, green) that were dissected from new born mice and cultured for 1 week and then were transfected with siRNA and treated with or without MPH for 3 days (scale bars, 15 μm). White boxes indicate the enlarged regions (*n* = 3 independent experiments for neurons, *n* = 2 independent experiments for NPCs and glial cells, above five fields containing at least 100 cells were analyzed per condition; two‐way ANOVA). (e–g) Representative microscopy images and its quantification of SA‐β‐gal signals in primary neurons (stained with β‐tubulin, green), NPCs (stained with SOX2, green), and glial (stained with GFAP, green) cells (scale bars, 25 μm for neuron, 50 μm for NPCs and glial cells). Quantification analysis of percentage of SA‐β‐gal‐positive cells (*n* = 3 independent experiments for neurons, *n* = 2 independent experiments for NPCs and glial cells, more than 100 cells were analyzed per condition; two‐way ANOVA). (h) Relative mRNA expression levels of *Ppp2r2c*, *Mcm2*, *Mcm5*, *Mcm6*, and *Top2a* in primary mouse neurons, NPCs, and glial cells determined by RT‐qPCR (*n* = 5 independent experiments for neurons and NPCs; *n* = 3 independent experiments for glial cells; unpaired two‐sided *t* test). Data are means ± SEM. *p < 0.05, **p < 0.01

We investigated the alterations induced by *Ppp2r2c* inhibition in mouse primary neural cell through analysis of glial cells, neural progenitor cells (NPCs), and neurons isolated from embryonic mouse brains (Figure 7a). Inhibition of *Ppp2r2c* in these three types of primary cells induced a potent DDR signaling as indicated by increased levels of cells positive for γH2AX foci (Figure 7b–d). Except in glial cells, *Ppp2r2c* downregulation also triggered cellular senescence, as demonstrated through a SA‐β‐gal assay (Figure 7e–g). Mirroring the transcriptomic changes observed in the adult brains of *ppp2r2c* ^*m1/m1*^ zebrafish (Figure 3a), the *Ppp2r2c* inhibition in mouse glial cells and progenitor cells triggered the overexpression of genes involved in replication (Figure 7h). In contrast, *Ppp2r2c* loss led to the downregulation of replication‐related genes in cultured primary mouse neurons (Figure 7h). Overall, these results reveal that Ppp2r2c connects the processes of DNA damage, replication control, and senescence in mouse neural cells.

### 2.10 PP2A activators have senomorphic properties

Finally, we investigated the capacity of a PP2A activator to act as a general geroprotective drug by testing whether MPH could attenuate the effects of DDR in cultured *ppp2r2c‐*compromised neural cells. DDR activation in glial cells, NPCs, and neurons was reduced with MPH treatment (Figure 7b–d), while the appearance of SA‐β‐gal‐positive cell was prevented by MPH treatment only in neurons (Figure 7e–g). Moreover, the upregulation of *mcm* genes in glial cells and NPCs was prevented by MPH treatment (Figure 7h). The capacity of MPH to reverse the increased level of γH2X, senescence, and *mcm* gene dysregulation in mouse primary neural cells after *ppp2r2c* downregulation mirrored the effects of the drug in the *ppp2r2c* ^*m1/m1*^ zebrafish brain.

## 3 DISCUSSION

This study provides evidence that natural aging is accompanied by a decreased level of PP2A activity in the brain and certain behavioral phenotypes in zebrafish (22 months) and mouse (14 months). The association between the reduction of PP2A activity and age‐related changes was demonstrated by the similar behavioral phenotype observed in young fish with a mutated brain‐specific isoform of the PP2A regulatory subunit (*ppp2r2c*), as well as by the reversal of the age‐related behavioral changes with PP2A activator treatment. Whether the behavioral effects of *ppp2r2c* loss described here are mechanistically related to the various human mental illnesses associated with *ppp2r2c* gene polymorphism(Backx et al., 2010; Jacob et al., 2012; Kimura et al., 2019; Xu et al., 2014) is an interesting question for future research.

In both *ppp2r2c*‐compromised adult and WT aged zebrafish brains, more neuronal (NeuN (+)) and non‐neuronal (NeuN (−)) cells exhibit senescence markers (γH2X, cell cycle checkpoints, SA‐β‐gal and SASP) as compared to WT adult and young zebrafish, respectively. This senescence phenotype is absent in fish treated with various PP2A activators, indicating that decreased PP2A in neural cells, whether due to the loss of *ppp2r2c* or natural aging, can lead to cell senescence. We also provide evidence that this senescence phenotype plays a role in the behavioral abnormalities observed in zebrafish. Indeed, they disappear with inactivation of the *tp53* and *cdkn1a* genes or treatment with ABT263, which is a classical senolytic agent that provokes the apoptosis of senescent cells. Notably, in both types of zebrafish, the senescence phenotype triggered by PP2A activity impairment specifically affects the OT region of the brain. This localization likely stems from the higher level of *ppp2r2c* mRNA expression in neural cells of the OT relative to other parts of the brain (Figure S7e,f). The behavioral consequences of this OT‐specific alteration might be explained by an impact of the visuomotor dysfunction leading to anxiety and hyperactivity, as the OT mediates the detection and the integration of visual information to generate behavior (Duchemin et al., 2022). As the neural tectal cells of *ppp2r2c*‐compromised zebrafish exhibit higher levels of pro‐inflammatory gene expression than WT zebrafish (Figure 3d), neuroinflammation effect could play a role in behavioral abnormalities related to PP2A decline.

An important finding of this study is that that PP2A activation has anti‐senescence properties. Since the DDR marker that we used (γH2AX) can be a substrate of PP2A (Chowdhury et al., 2005; Ferrari et al., 2017), the decreased γH2AX level upon PP2A activation cannot simply be interpreted as a decreased level of physical DNA damages but also according to the role of γH2AX in cell‐cycle checkpoint activation and senescence. That PP2A activation can lead to γH2AX dephosphorylation does not exclude other effects of PP2A in modulating DDR and senescence (Ramos et al., 2019).

Our results link PP2A, brain aging, neural senescence, and age‐related behavioral changes. We propose that the age‐related decrease in PP2A activity increases the level of DDR leading to neural senescence, neuroinflammation, and behavioral changes. An important finding of this study is that drugs with PP2A activation activity can prevent age‐related cognitive decline. In particular, we revealed that the classical psychostimulant MPH (Ritalin ^R^) activates PP2A activity in aged brains, attenuates the DDR, and reduces the abundance of SA‐β‐gal‐positive neurons. This mechanism can explain the findings of recent clinical studies showing that treatment with MPH improves the outcomes of age‐related dementia (Padala et al., 2018; Scherer et al., 2018). As the long‐term use of MPH may cause side effects, including cardiac attack (Shin et al., 2016), the finding that MPH is effective even with a very short period of application, in terms of increasing PP2A activity, reducing the DDR and neural cell senescence, and countering age‐related behavioral changes, suggests that it has promise as an anti‐aging intervention when used intermittently during short periods.

## 4 METHODS AND MATERIALS

Experimental details can be found in the Methods and Materials S1. Details on chemical regents, antibody, and PCR primers can be found in the Methods and Materials S1 and Tables S3 and S4.

## AUTHOR CONTRIBUTIONS

JY and EG designed the experiments. JX, KHC, SYG, YLY, BW, CCW, and LXC performed the experiments. JY, YML, and EG analyzed the data. EG and JY wrote the paper.

## CONFLICT OF INTEREST

The authors have declared that no conflict of interest exists.

## Supporting information


Appendix S1

Table S1

Table S2

Table S3

Table S4

Figures S1‐S10

Appendix S2

## Data Availability

Source data are available for Figures 1, 2, 3, 4, 5, 6, 7 and S1–S10. All data and information relevant to this study are available from the corresponding author upon reasonable request. RNA‐seq data had been deposited in the Gene Expression Omnibus (GEO) with accession number GSE151307.
