# Supplementary material for: Protein phosphatase 2A activators reverse age‐related behavioral changes by targeting neural cell senescence
Source: Aging Cell. 2023 Jan 16;22(3):e13780. doi: 10.1111/acel.13780 (PMC10014060; doi:10.1111/acel.13780)
Supplement: Supplementary file 1 — Appendix S1 [file ACEL-22-e13780-s002.docx]

**Method and Materials**

**Subject and drug treatment.** These experiments were performed in the Zebrafish Tübingen strain. The zebrafish was raised and maintained under standard conditions as described previously(Kimmel, Ballard, Kimmel, Ullmann, & Schilling, 1995). All experiments were performed in accordance with the guidelines of the Animal Care and Use Committee of Shanghai Jiao Tong University. Both 14 and 3-month-old C57 B6/J mice were produced and maintained in the animal facility of Ruijin Hospital, Medical School of Shanghai Jiaotong University. The protocol was approved by the Committee on the Ethics of Animal Experiments. For zebrafish All drug solutions were freshly prepared with PBS (MPH 1.08 mg/kg, valproate 300 mg/kg, aripiprazole 0.6 mg/kg, ziprasidone 1.6 mg/kg, DT-061 and FTY720 5mg/kg). The zebrafish was given food soaked in the drug solutions for 3 days and behavioral analysis was performed on forth day. Both 14 and 3-month-old C57 B6/J mice were fed with MPH (12.3mg/kg/d) by gavage for 14 days in total. Psychiatry drugs’ dosage were determined by clinical prescription used in human and converted by animals’ weight. DT-061 and FTY720 treatment were based on the published study(Kauko et al., 2018; Vicente et al., 2020).

**Generation of CRISPR-Cas9 mutant zebrafish.** *ppp2r2c*-mutant zebrafish were generated using the CRISPR-Cas9 system, in which a guide RNA targeting exon 9 of *ppp2r2c* (sgRNA: 5′-GGGCCCAGTGGAGACATACC-3′) and exon 2 of *cdkn1a^p21^* (sgRNA: 5′- GGTAATGGGCCGACTAGG -3′) were designed using ZiFiT Targeter software (http://zifit.partners.org/ZiFiT). The guide RNA was synthesized by cloning annealed oligonucleotides into the sgRNA vector as described previously. The founder embryos (F0 generation) were raised to 3 months old and outcrossed with WT zebrafish to obtain potential F1 indel mutations. PCR amplification and sequencing were performed on the genomic DNA isolated from the tails of F1 zebrafish to identify *ppp2r2c* mutants (primers for genotyping: fwd 5′-CAGGCCACAGTGTTGAAGAT-3′; rev 5′-GTGCTGAAGAGCTGCACTAA-3′) and *cdkn1a^p21^* mutants (primers for genotyping: fwd 5′- TCTGTCTGAGGTATTGGGTG -3′; rev 5′- GAGTGCAGCACATCGTTC -3′).

**Primary cell isolation and transfection.** Primary neurons were isolated from the cortex of newborn C57BL/6 mice and cultured in Neurobasal-A™ medium (#10888022; Gibco) supplemented with B27 supplement (#17504-044; Gibco) and L-glutamine (#07100; Stemcell Technologies). Primary NPCs were isolated from the hippocampus of newborn C57BL/6 mice and cultured as neurospheres in NeuroCult™ Basal Medium (#05700; Stemcell Technologies) with proliferation supplement (#05701; Stemcell Technologies) containing 20 ng/mL EGF (#78006.1; Stemcell Technologies) and 10 ng/mL bFGF (#78003.1; Stemcell Technologies). Primary glial cells were isolated from the cortex of newborn C57BL/6 mice and cultured in DMEM/F-12, HEPES (#11330032; Gibco) supplemented with 10% fetal bovine serum (FBS; #10099141; Gibco). The ON-TARGET plus Mouse *Ppp2r2c* siRNA SMART pool (#L-055606-01; Dharmacon) was used for transfection of primary cells according to cell type. For glial cells, transfections were performed using a Lipofectamine 2000 kit (#11668027; Thermo Fisher Scientific) in accordance with the manufacturer’s instructions, and the culture medium was replaced 6 hours after transfection. For neurons and neural stem cells, transfections were performed using X-tremeGENE HP DNA Transfection Reagent (Roche; #06 366 236 001) in accordance with the manufacturer’s instructions.

**RNA-seq analysis.** Sequencing was performed by BGI. Illumina HiSeq Xten 2 ×150-bp paired-end was used for sequencing. More than 40 million raw reads were obtained for each sample. Raw RNA-seq reads were filtrated by SOAPnkue with default setting to remove adapters and low-quality reads and the remaining reads were trimmed into 100bp for downstream analysis. Reads were aligned using STAR v2.6.1d on zebrafish zv10 release with parameter–quantMode GeneCounts. Differential expression analysis was performed using DESeq2 R package and differential expression genes (p value < 0.05) were used for downstream analysis. Enriched pathways were identified by ingenuity pathway analysis software (IPA) with default setting.

**RT-qPCR.** Total RNA from adult zebrafish tissue was extracted using TRIzol reagent (Ambion) and reverse transcribed using the PrimeScript RT reagent kit (TaKaRa) according to the manufacturers’ protocols. RT-qPCR analysis was performed using the QuantStudio Dx Real-Time PCR Instrument (ABI) with SuperRealPreMix Plus (TianGen). The primers used in this study are shown in Supplementary Table S3.

**PP2A phosphatase assay.** Homogenates prepared from zebrafish brains, mouse frontal and temporal lobes were placed in 20 mM imidazole–HCl, 2 mM EDTA, 2 mM EGTA, pH 7.0 with 10 μg/mL each of aprotinin, leupeptin, and pepstatin, 1 mM benzamidine, and 1 mM phenylmethylsulphonyls fluoride and lysis by adding 1% NP-40. The assays were then performed using PP2A Immunoprecipitation Phosphatase Assay Kit (Millipore) in accordance with the manufacturer’s instructions. The absorbance at a wavelength of 650 nm (A_650_) was measured in a microtiter plate reader (BioTAK).

**Neurotransmitter’s assay.** Brains of adult fish at 6-month-old were dissected out and fast frozen in liquid nitrogen. Measurement and analysis of 23 endogenous neurotransmitters concentration by ultra-performance liquid-chromatography tandem mass spectrometry (UHPLC-MS/MS) was performed by Shanghai Biotree biotech Co.Ltd. A standard solution of metabolites was prepared and subjected to UHPLC-MS/MS analysis to get calibration curves. The levels of metabolites were quantified according to calibration curve. The UHPLC separation was carried out using an Agilent 1290 Infinity II series UHPLC System (Agilent Technology), equipped with a Waters ACQUITY UPLC HSS T3 column (100 × 2.1 mm, 1.8 μm). An Agilent 6460 triple quadrupole mass spectrometer (Agilent Technology), equipped with an AJS electrospray ionization (AJS-ESI) interface, was applied for assay development. Typical ion source parameters were: capillary voltage = +4000/-3500 V, Nozzle Voltage = +500/-500 V, gas (N_2_) temperature = 300 ^o^C, gas (N_2_) flow = 5 L/min, sheath gas (N_2_) temperature = 250 ^o^C, sheath gas flow = 11 L/min, nebulizer = 45 psi. Agilent MassHunter Work Station Software (B.08.00, Agilent Technologies) was used for MRM data acquisition and processing.

**Reactive oxygen species (ROS) assay**. Cells were isolated from zebrafish brain by single cell suspension preparation. Then cell suspension was spined on slice at 600rpm. The ROS assays were then performed using the Reactive Oxygen Species Assay Kit (Beyotime) in accordance with the manufacturer’s instructions. Imaging was taken by Zeiss Z1 microscope.

**Immunofluorescence imaging of brain slices.** Zebrafish tissues were separated and fixed with 4% PFA overnight in 4 °C follow by dehydrate with 30% sucrose overnight. Tissues were then embedded in OCT and sectioned at 5 μm. Frozen sections and chamber slides were fixed in 4% PFA for 30 minutes and then permeabilized and blocked with 0.5% Triton X-100, 2% FBS in 1× PBS for 1 hour at room temperature. Hybridization with primary antibodies was performed at 4°C overnight followed by washing three times with 0.1% Tween-20 in PBS. Hybridization with corresponding secondary antibodies (Invitrogen) was performed at 37°C for at least 2 hours. Finally, the slices were incubated with 1× DAPI for 5 minutes at room temperature. Imaging was performed using a confocal laser scanning microscope (SP8; Leica). The antibodies used in this study are shown in Supplementary table S4.

**RNAscope® in situ hybridization combined with immunofluorescence.** RNA-Protein Co-detection Ancillary Kit (Advanced Cell Diagnostics) and RNAscope® Multiplex Fluorescent Reagent Kit v2 (Advanced Cell Diagnostics) were used accordance with the manufacturer’s instructions. Briefly, frozen tissue sections were moved from -80 °C then washed with PBS for 5 min. Slices were baked in a dry oven for 30 min at 60 °C and fixed in 4% PFA at 4 °C for 15 min. Sections were dehydrated then incubated with RNAscope® Hydrogen Peroxide for 10 min at room temperature. After washing twice with distilled water, manual target retrieval was performed by boiling the sections (100 to 103 °C) in 1× Target Retrieval Reagents for 5 min, and then incubated with primary antibody at 4 °C overnight. After three washes in PBS-T, sections were incubated with RNAscope® Protease Plus at 40 °C for 30 min in a HybEZ hybridization oven (Advanced Cell Diagnostics). Hybridization with target probes (DR-cdkn2a Cat. 431681; DR-cdkn1a-C3 Cat. 457401-C3; DR-tnfa Cat. 575111; DR-cxcl8a-C2 Cat. 522981-C2; DR-ppp2r2ca Cat. 1079831-C1. Advanced Cell Diagnostics) was carried out incubating the slides at 40 °C for 2 h, and then the slides were incubated at 40 °C with the following reagents: Amplifier 1 (30 min), Amplifier 2 (30 min), Amplifier 3 (15 min); HRP-C1 (15 min), TSA® Plus fluorophore for channel 1 (fluorescein, cyanine 3, or cyanine 5, PerkinElmer; 1:1000; 30 min), HRP blocker (15 min); HRP-C2 or HRP-C3 (15 min), TSA® Plus fluorophore for channel 2 (30 min), HRP blocker (15 min). After each hybridization step, slides were washed twice with Wash Buffer at room temperature. After hybridization, slices were incubated with fluorescently labelled second antibody at room temperature for 1 hour and then stain with DAPI. Slices were mounted in Vectashield and examined under fluorescent confocal microscope (SP8, Leica). The antibodies used in this study are shown in Supplementary table S4.

**Western blot.** Protein lysates were prepared by RIPA (50mM Tris (pH 7.4), 150mM NaCl, 1% Triton X-100, 1% sodium deoxycholate, 0.1% SDS) complemented with DMSF (Beyotime Biotechnology), phosphatase and protease inhibitors (Roche). Protein concentration was quantified by Pierce^TM^ BCA Protein Assay Kit (Thermo). Protein was loaded onto SurePAGE 4-20% Bis-Tris gradient gels (Genscript). Sample was transferred onto Immobilon-P PVDF 0.22μm membranes (Millipore) using Trans-Blot Turbo Transfer System (BioRad). Membranes were blocked in 5% NON-Fat Powdered Milk (Sangon) with TBST (0.1% Tween-20 in TBS) for 1 hour at room temperature (RT). Hybridization with primary antibodies (shown in Supplementary information. Table S4.) was performed at 4ºC overnight followed by TBST washing three times. Hybridization with corresponding second antibodies was performed at 37ºC for 2 hours followed by TBST washing three times. Imaging was detected using AI600 (GE) and processed using ImageJ software. The antibodies used in this study are listed in Supplementary Table S4.

**Zebrafish behavioral assays.**

An adult fish was placed in a standard mating tank (21 × 10 × 7.5 cm) containing system water to a depth of 6 cm and allowed to acclimatize for 15 minutes and then transferred to an automated observation and video tracking system (ZebraLab; Viewpoint Life Sciences). **Light-dark transition assay.** An adult fish was placed in the dark and allowed to acclimatize for 15 minutes. The light was then turned on for 30 s, and the activity was recorded using the ZebraLab quantization software module. Quantization tests focus on the activity of zebrafish which represent the global amount of movement of zebrafish in the tanks and its frequency. The software can automatically record the position of the zebrafish and compare the previous position with the new position. The changed pixels are regarded as the activity. We used the wild-type fish getting two thresholds’ values (high threshold value is 100 and freezing value is 20). The high threshold value indicates that the pixel change of 95% movements is below this value. The freezing value means the pixel change is below this value when the fish stop moving. If the value of moving surface above the high threshold, the activity will be recorded as high activity and the software will automatically record the duration of the activity. (Viewpoint Life Sciences). **Mirror attack test.** A mirror was placed outside of the end of the tank, and the attacking behavior of the fish was monitored continuously for 5 minutes using the ZebraLab tracking software module (Viewpoint Life Sciences). **Open-field test.** The movement of the zebrafish was monitored continuously for 30 minutes. The time spent in the central area (30% of the total area) was recorded and analyzed using Video tracking software (Viewpoint Life Sciences). **Sleep test.** The light of automated observation and video tracking system (ZebraLab; Viewpoint Life Sciences) was turned off after 8 :30 pm and the activity was recorded using the ZebraLab quantization software module (Viewpoint Life Sciences). We regarded freezing activity as sleep. If the value of moving surface lower than the freezing threshold, the activity will be recored as freezing activity and the software will automatically record the duration of the activity. **Social contact.** The distance between two fish shorter than the length of thresholds to be considered as a social contact. Two adult fish was placed in a mating tank (21 × 10 × 7.5 cm) containing system water to a depth of 6 cm and allowed to acclimatize for 15 minutes and then monitored by an automated observation and video tracking system (ZebraLab; Viewpoint Life Sciences) for 60 minutes. The ZebraLab social software module automatically recorded the number of times the two fish was close to each other.

**Mice behavioral assays.**

**Morris Water Maze.** Morris Water Maze was conducted in a round blue pool and a platform which are 120cm and 10cm in diameter respectively. The round area was separated into 4 quadrants and a platform area. 4 pieces of paper in different shapes were hanged around the pool. The experiments took 6 days in total. During the first 5 days, the platform was set in a fixed position, then mice were lowered in to the pool with their heads heading to the wall from every quadrant. The latency, the distance before mice getting onto the platform were recorded by software from Shanghai XinRuan Information Technology Co.,Ltd, as the judgement for their learning ability. On the 6^th^ day, the procedure was repeated after removing the platform. The frequency mice crossed the area of the platform, the time spent in the correct quadrant and the average proximity to the platform location were recorded as the judgement for their memory. **Light/dark transition test.** We used an apparatus consisting of a light, open topped, opaque, Plexiglas box (25 × 25 × 30 cm) connected to a dark, closed topped, opaque, Plexiglas box (20 × 20 × 30cm). The animals were placed in the illuminated box and left free to move from the light to the dark chamber through a connecting door (12 × 5 cm) for 5 minutes. The light box was illuminated by a desk lamp. A mouse was considered in the light chamber if the four paws were all in the light box. The number of transitions between the light and the dark chamber was collected.

**Cellular apoptosis assay****.** For sections of zebrafish brain, heart and kidney, the apoptosis assay was performed with in Situ Cell Death Detection Kit, TMR red (Roche). For MRC5 cells, the apoptosis assay was performed with TUNEL Apoptosis Detection Kit (Alexa Fluor 488) (Yeasen). The assays were performed following the manufacturer’s instruction. The images were taken by Leica SP8 confocal laser scanning and Zeiss Z1 microscope.

**Senescence-associated-β-galactosidase (SA-β-gal) assay.** SA-β-gal stain was performed using Senescence β-galactosidase Staining Kit (Beyotime). The assays were performed following the manufacturer’s instruction. Images were taken with Zeiss A2 microscope.

**Chemical reagents.** MPH (Ritalinic acid hydrochloride solution, sigma #R-011); ABT263 (MedChemExpress #923564-51-6); DT-061 (MedChemExpress #1809427-19-7); FTY720 (Selleck # 162359-56-0).

**Quantification and statistical analysis.** The data were analyzed by GraphPad Prism software. Data used in this study are pass normality and lognormality Tests. The statistical significance between two groups was determined using unpaired Student’s t-test, with tow-tailed P value. Among three or more groups, one-way analysis of variance followed by Bonferroni’s multiple comparison test or Dunnett’s multiple comparison test was used for comparisons. Values of P<0.05 were considered statistically significant. Absence of statistical annotation means non-significance.

**Reference**

Kauko, O., O’Connor Caitlin, M., Kulesskiy, E., Sangodkar, J., Aakula, A., Izadmehr, S., . . . Westermarck, J. (2018). PP2A inhibition is a druggable MEK inhibitor resistance mechanism in KRAS-mutant lung cancer cells. *Science Translational Medicine, 10*(450), eaaq1093. doi:10.1126/scitranslmed.aaq1093

Kimmel, C. B., Ballard, W. W., Kimmel, S. R., Ullmann, B., & Schilling, T. F. (1995). Stages of embryonic development of the zebrafish. *Developmental Dynamics, 203*(3), 253-310. doi:<https://doi.org/10.1002/aja.1002030302>

Vicente, C., Arriazu, E., Martínez-Balsalobre, E., Peris, I., Marcotegui, N., García-Ramírez, P., . . . Odero, M. D. (2020). A novel FTY720 analogue targets SET-PP2A interaction and inhibits growth of acute myeloid leukemia cells without inducing cardiac toxicity. *Cancer Letters, 468*, 1-13. doi:<https://doi.org/10.1016/j.canlet.2019.10.007>
