# Supplementary material for: Protein phosphatase 2A activators reverse age‐related behavioral changes by targeting neural cell senescence
Source: Aging Cell. 2023 Jan 16;22(3):e13780. doi: 10.1111/acel.13780 (PMC10014060; doi:10.1111/acel.13780)
Supplement: Supplementary file 4 — Table S3 [file ACEL-22-e13780-s001.docx]

**Supplementary Table 3. RT-qPCR primers used in this study**

| RT-qPCR | Primer-Fwd | Primer-Rev | Species |
| --- | --- | --- | --- |
| *ppp2r2c(p1)* | ATCACAGCTTCCTCCGAGAC | CCGATTCTCCCAGCTCTTCA | Zebrafish |
| *ppp2r2c(p2)* | GAAAAGGGCCCAGTGGAGA | GTTTACTGCTCTCACGCCAG | Zebrafish |
| *ppp2r2c(p3)* | TGAGCTGTGGTACGTGGAAT | CGGGAAGCACGTGATAGGAA | Zebrafish |
| *cdkn2a/b* | GGTAACGCACCTTTGGCTCT | GCGCCGAAGCGGATTAAAAG | Zebrafish |
| *cdkn1a* | TCACAGATTTCTACCAAGCCAAGA | CGAATGCAGCTCCAGACAGA | Zebrafish |
| *il-1β* | TCCGCTCCACATCTCGTACT | AACCGGGACATTTGACGGAC | Zebrafish |
| *il6* | AGCAGGAATGGCTTTGAAGGG | GTCAGGACGCTGTAGATTCGC | Zebrafish |
| *il8* | GTAGATCCACGCTGTCGCTG | TACAGTGTGGGCTTGGAGGG | Zebrafish |
| *tnfα* | AGACCTTAGACTGGAGAGATGAC | CAAAGACACCTGGCTGTAGAC | Zebrafish |
| *mmp9* | GTTTCTGGTTCTGGGCACCT | TAGCATTGGAGATGACCGCC | Zebrafish |
| *gadd45a* | AACGTGGTCTTGTGTCTGCTG | GAGGTCCATCGACTCTCCTCC | Zebrafish |
| *mcm2* | TCGCAAGTATTGGACCCTTCA | CACCTTGTGCTTTCCACCTG | Zebrafish |
| *mcm5* | AACTCGCGCTGCTCTGTATT | GCCAAAGTCATGTCTCGCTG | Zebrafish |
| *mcm6* | AAGCCAGTTCCTCAAGCATGT | GTCAATGCAGCAAACACCGT | Zebrafish |
| *top2a* | AGCACATCAAAGGAGGCCAA | TAAAGGCCAGGGTGATAGCC | Zebrafish |
| *hpdb* | AGCACGAGGAAGGCAAATTCA | TCTCCAGTCCACGATACGCC | Zebrafish |
| *ifit10* | GGAGGCTGAAAGCTGGGTAG | CCTCGGGCATGGTGACTTTG | Zebrafish |
| *hipk1a* | TGTGTGCCCTCCTACCATCC | GTTTTGGGGCACTATGGGCA | Zebrafish |
| *β-actin* | GGGTATGGAATCTTGCGGTATC | CTTCATGGTGGAAGGAGCAA | Zebrafish |
| *Ppp2r2c* | GATTACCGAACGAGACAAGAGG | GAGATGGAGTTGATGTGGTAGG | Mouse |
| *β-actin* | AGAGCTATGAGCTGCCTGA | GGCATAGAGGTCTTTACGGATG | Mouse |
| *Mcm2* | AACACATATGGCGTGGAGCC | CCGTTGCCATGGACTCCTTC | Mouse |
| *Mcm5* | GGAGGAATTTCGTCGCCTGG | AGTGAGTCCATCTGGGAGCC | Mouse |
| *Mcm6* | TCAATGGTCATGCCGACAGC | ATTCAGCAAAGCCCAGCCTC | Mouse |
| *Top2a* | GCCTTGAGTGCTCGTGTCTC | TTGCTTTCTTGCTCGTGGCA | Mouse |
