# Supplementary material for: Protein phosphatase 2A activators reverse age‐related behavioral changes by targeting neural cell senescence
Source: Aging Cell. 2023 Jan 16;22(3):e13780. doi: 10.1111/acel.13780 (PMC10014060; doi:10.1111/acel.13780)
Supplement: Supplementary file 5 — Table S4 [file ACEL-22-e13780-s003.docx]

**Supplementary Table 4. Primary antibody used in this study**

| Primary antibody | Source | Identifier | Usage |
| --- | --- | --- | --- |
| Anti-γ-H2AX | GeneTex | Cat# GTX127342 | IF 1 :400 |
| Anti-NeuN | millipore | Cat# MAB377 | IF 1 :50 |
| Anti-SOX2 | GeneTex | Cat# GTX124477 | IF 1 :400 |
| Anti-GFAP | GeneTex | Cat# GTX128741 | IF 1 :400 |
| Anti-γ-H2AX | abcam | Cat# ab26350 | IF 1 :100 |
| Anti-γ-H2AX | abcam | Cat# ab2893 | IF 1 :300 |
| Anti-βIII Tublin | abcam | Cat# ab78078 | IF 1 400: |
| GAPDH | Yeasen | Cat# 30202ES40 | WB  1 :2000 |
| Anti-NeuN | millipore | Cat# ABN78 | IF 1 :400 |
| Anti-NeuN | abcam | Cat# ab177487 | IF 1 :40 or 1 :400 |
| Anti-PP2A, C subunit | millipore | Cat# 05-421 | WB 1 :2000 |
