# Supplementary material for: Protein phosphatase 2A activators reverse age‐related behavioral changes by targeting neural cell senescence
Source: Aging Cell. 2023 Jan 16;22(3):e13780. doi: 10.1111/acel.13780 (PMC10014060; doi:10.1111/acel.13780)

Supplementary Fig. 1

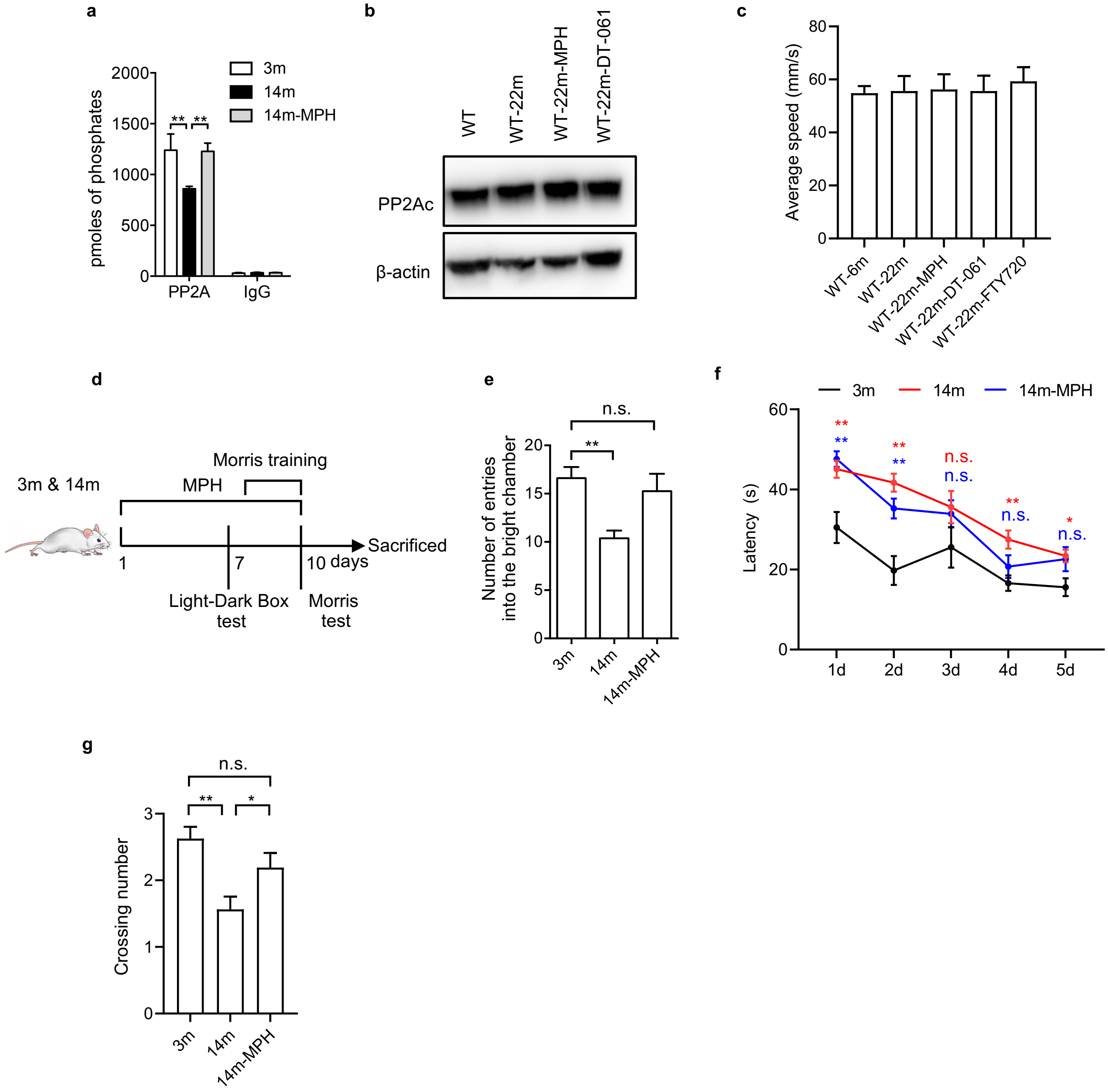

Supplementary Fig. 2

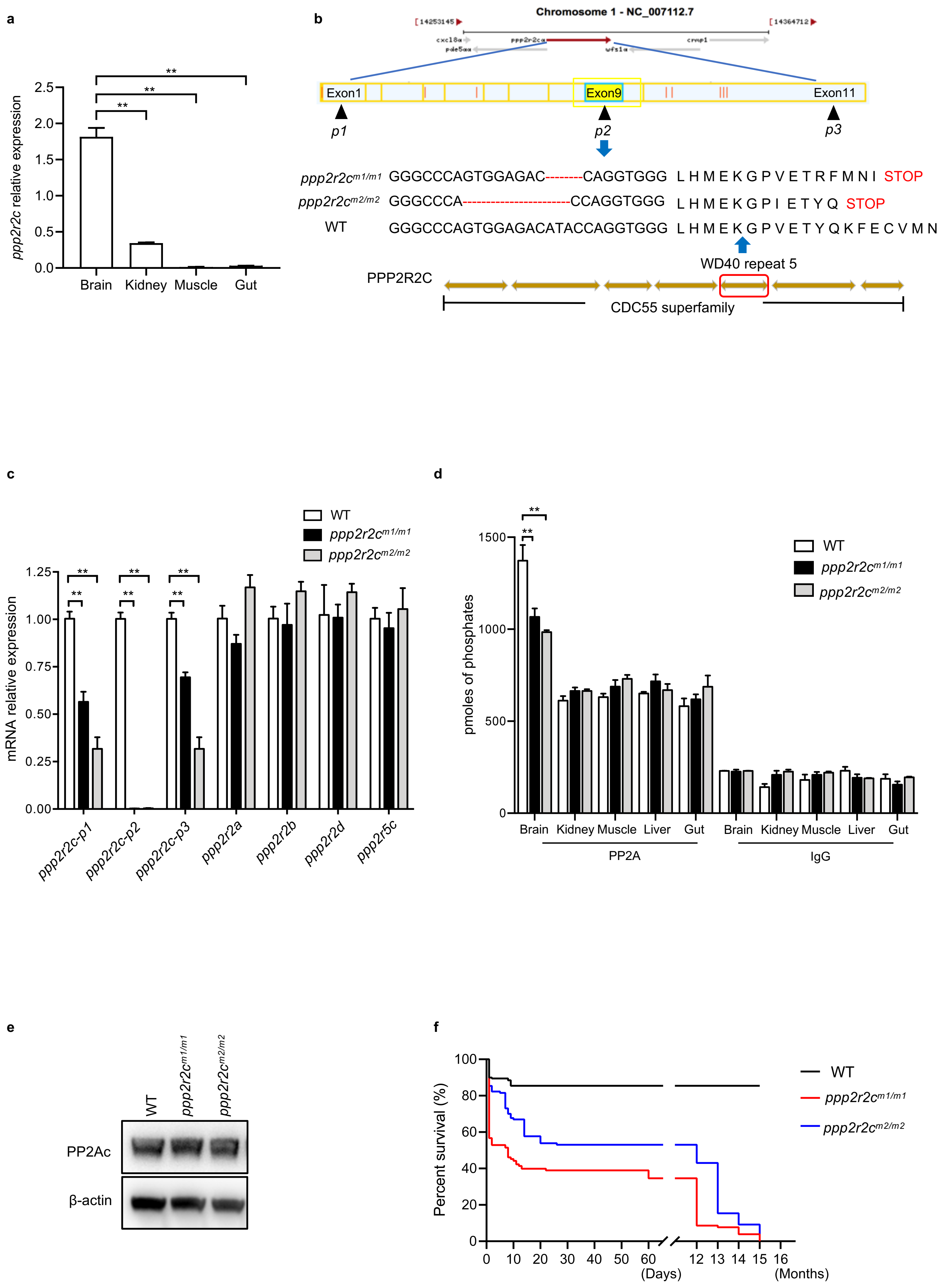

Supplementary Fig. 3

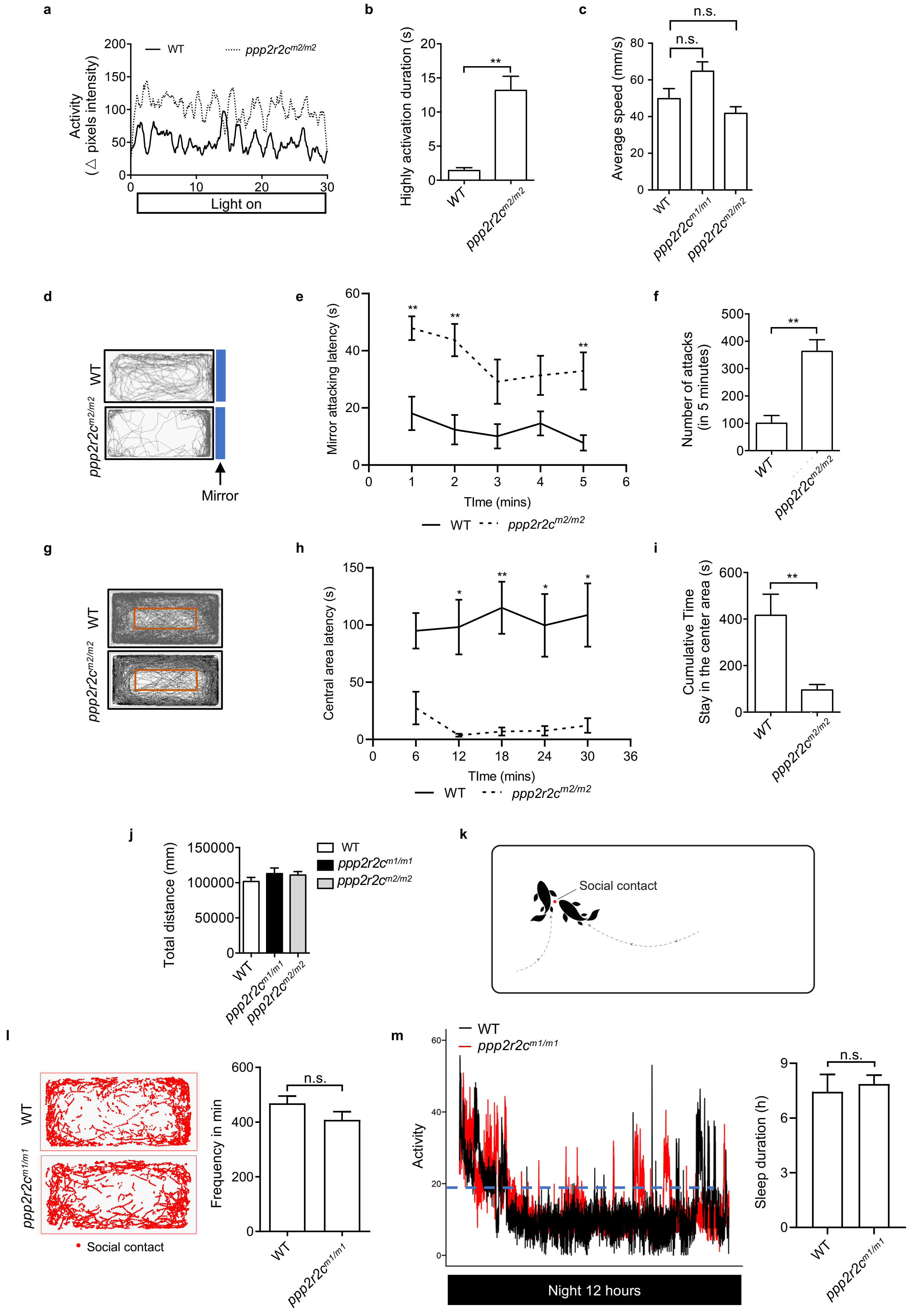

Supplementary Fig. 4

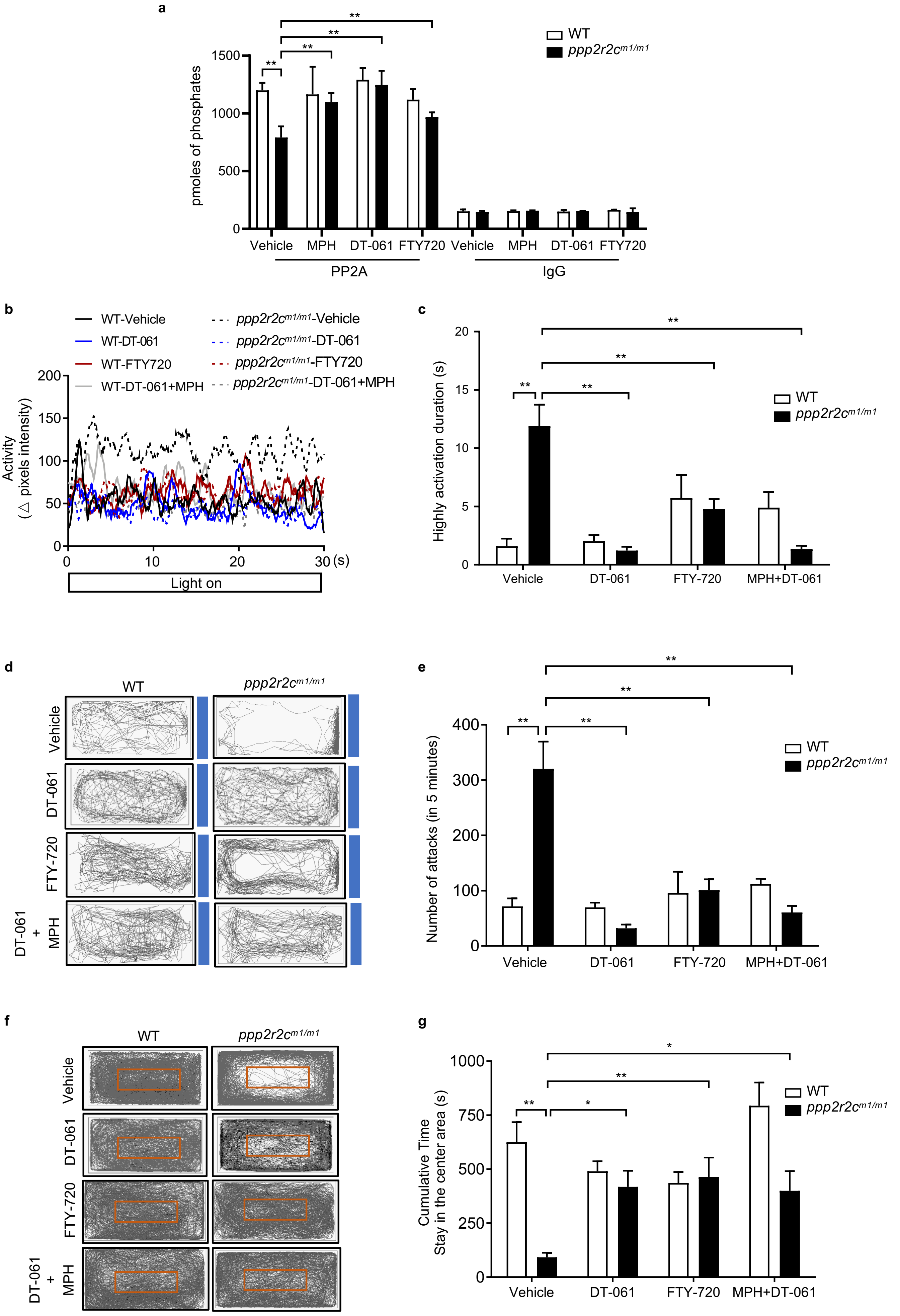

Supplementary Fig. 5

a

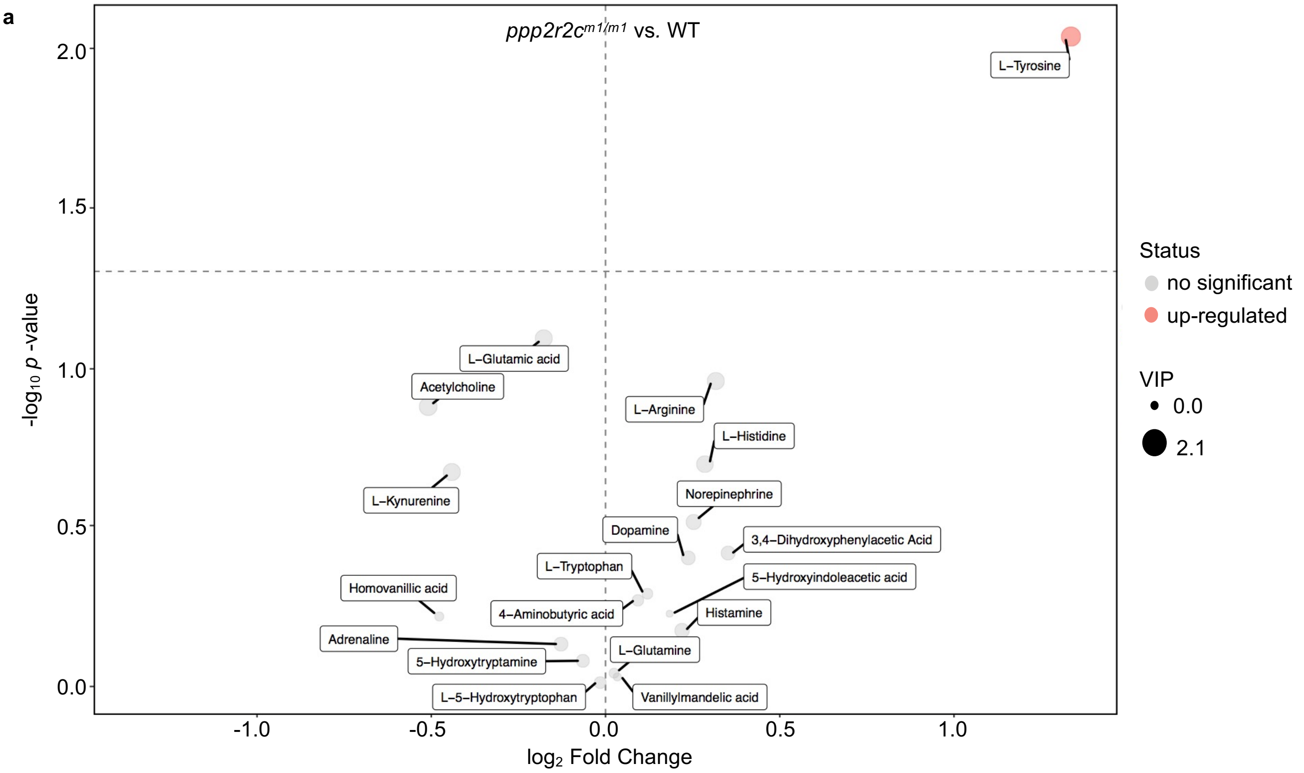

Supplementary Fig. 6

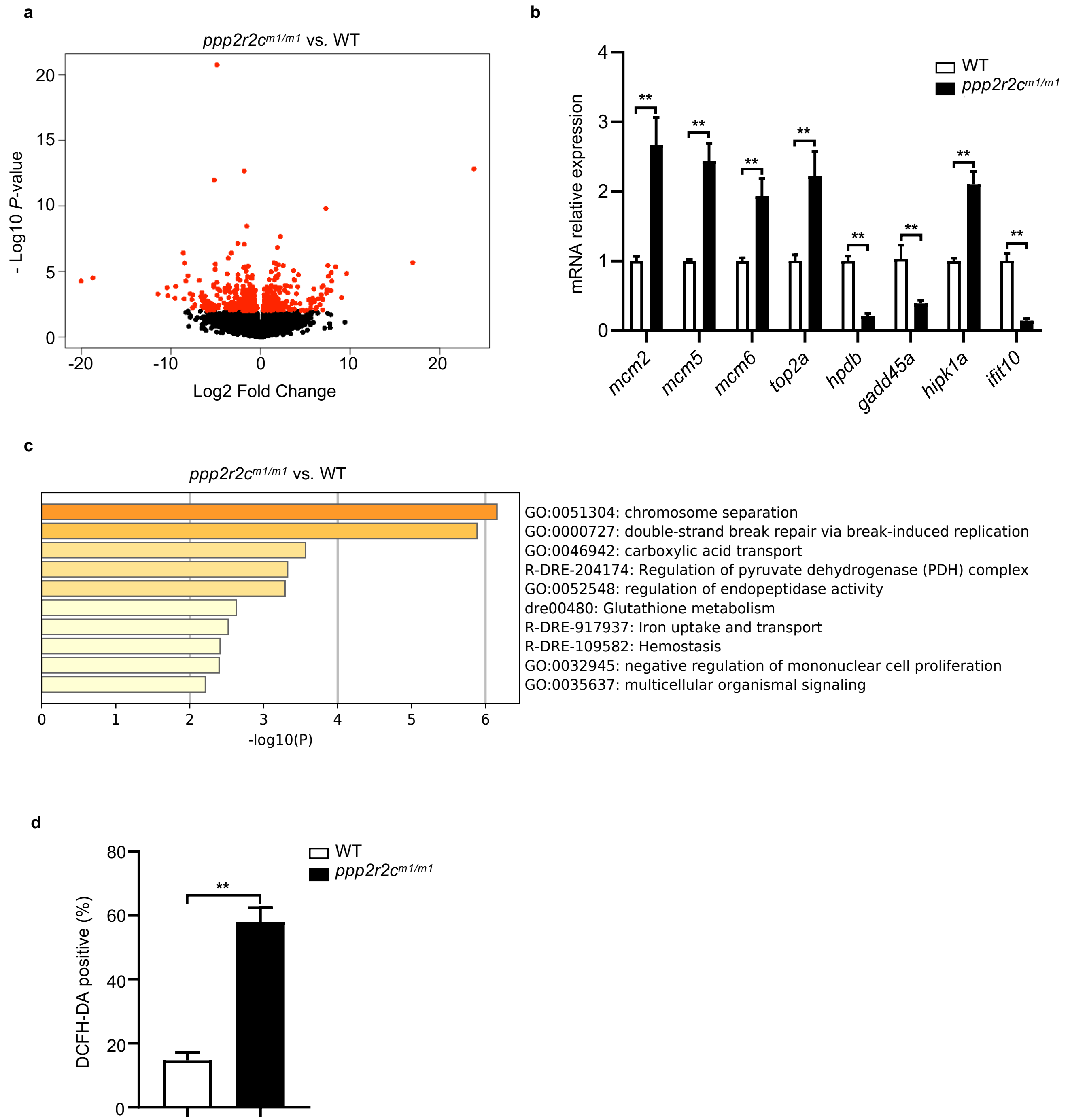

Supplementary Fig. 7

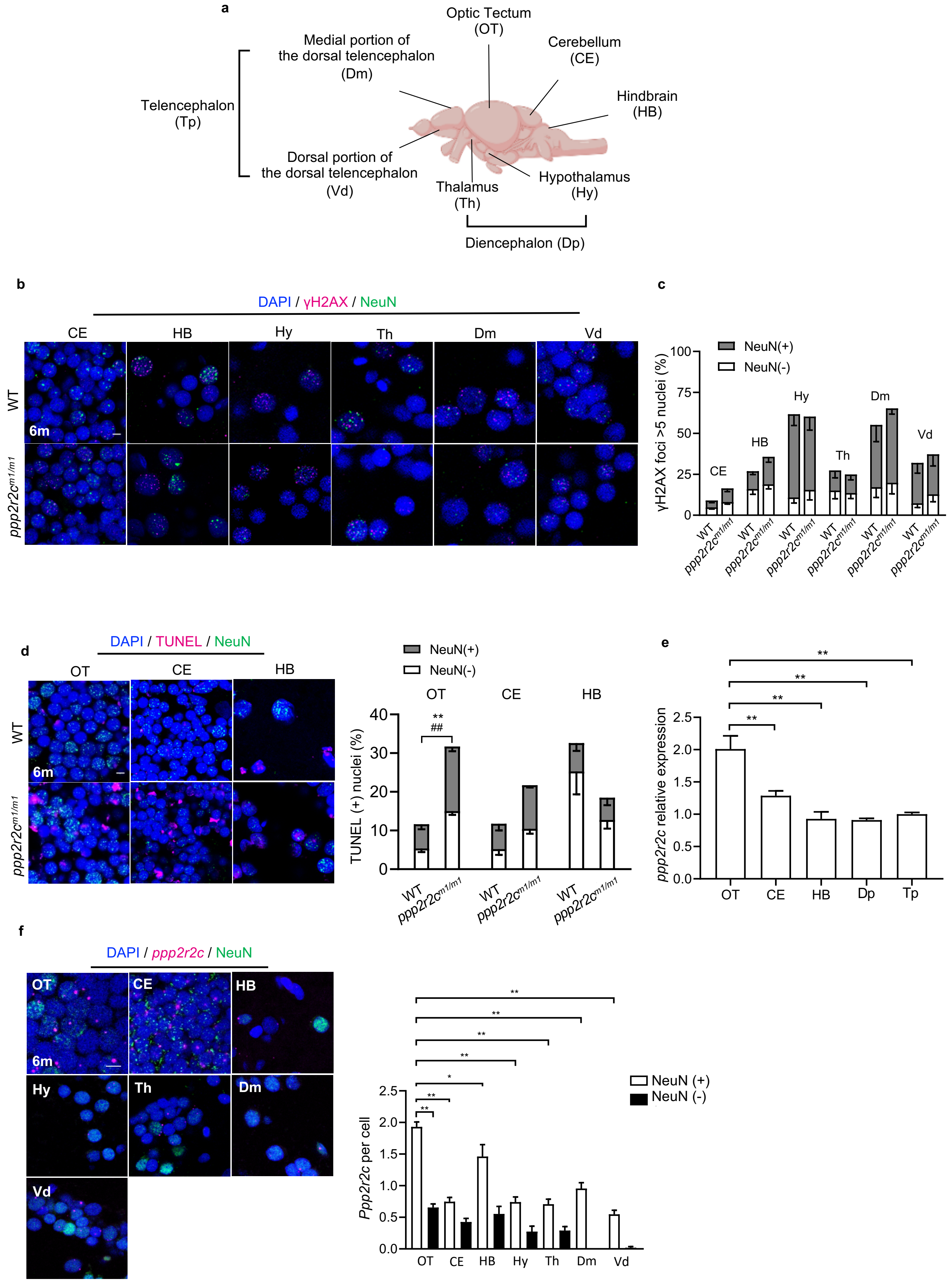

Supplementary Fig. 7

g

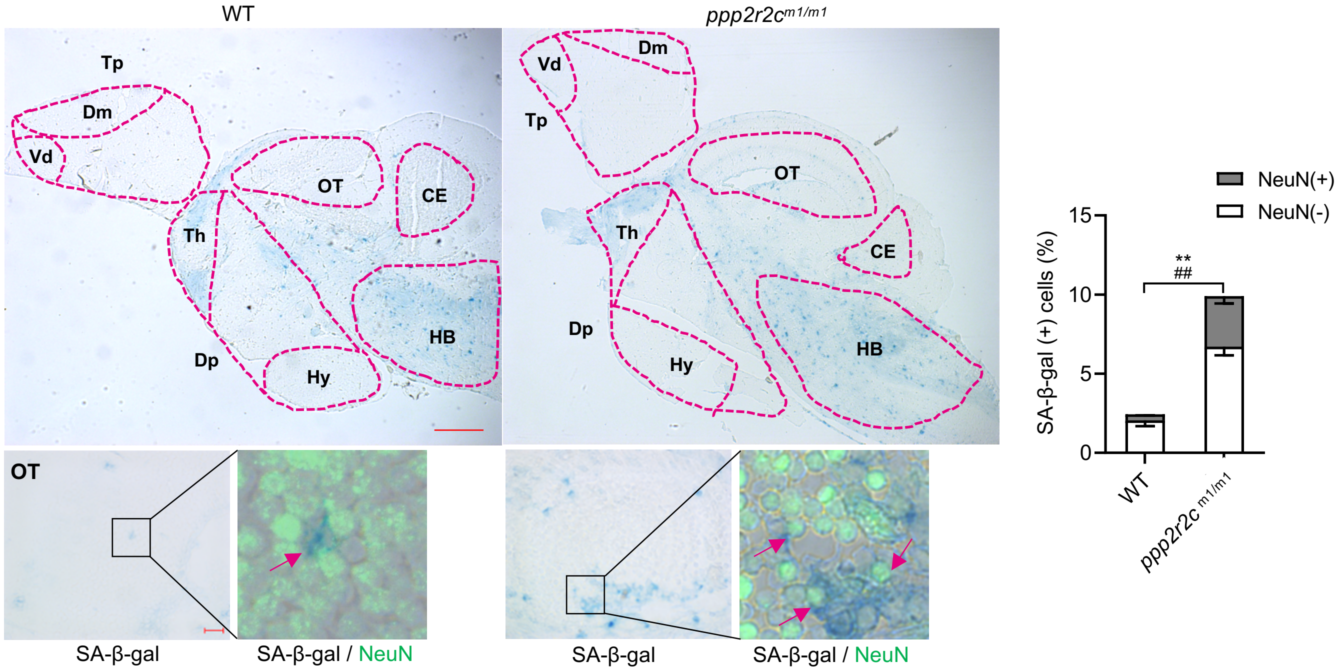

h

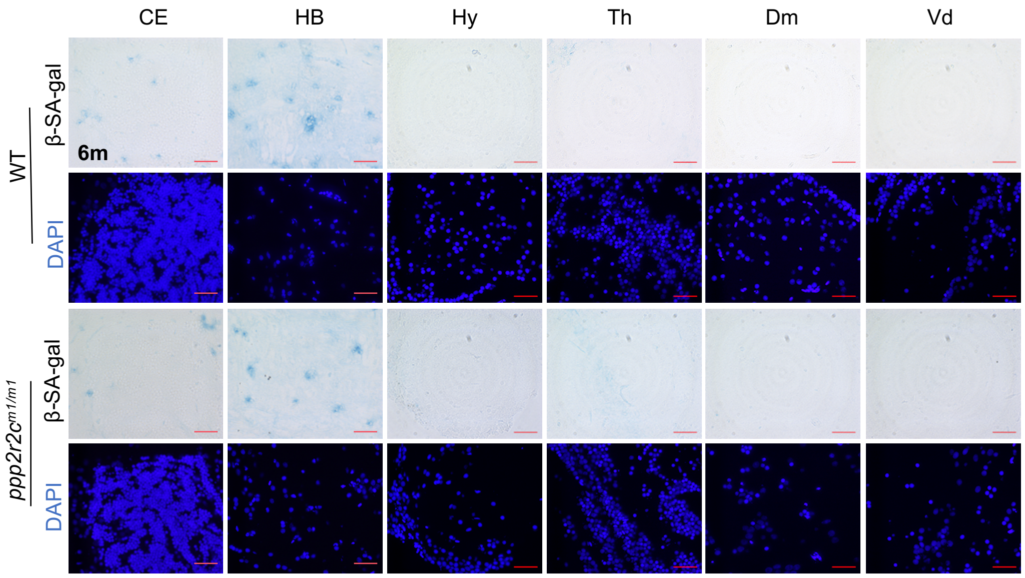

i

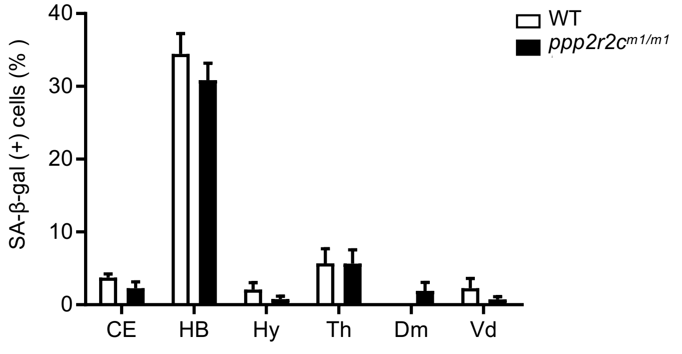

Supplementary Fig. 7

j

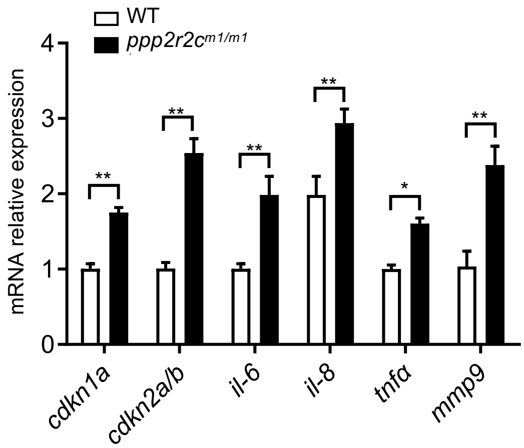

k

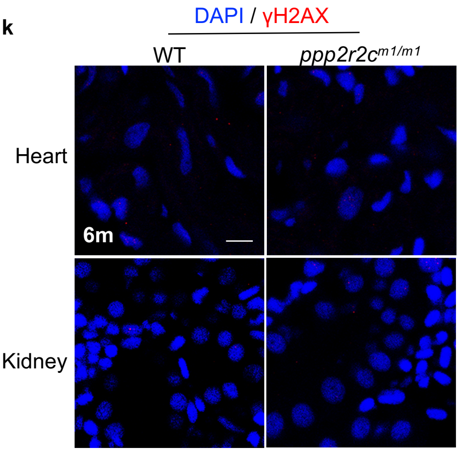

l

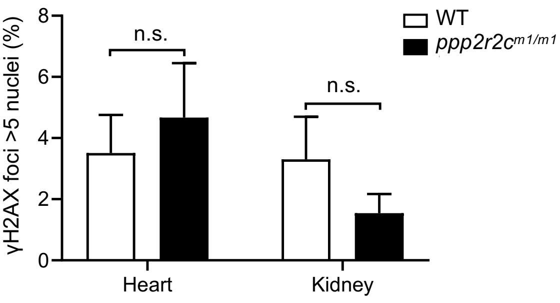

m

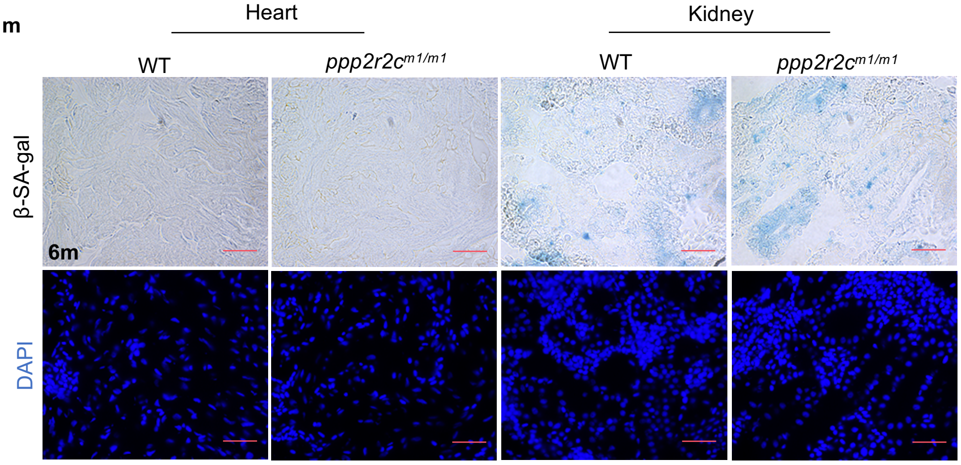

n

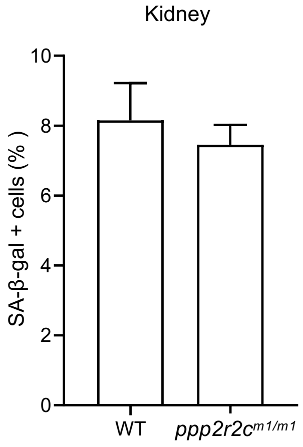

Supplementary Fig. 8

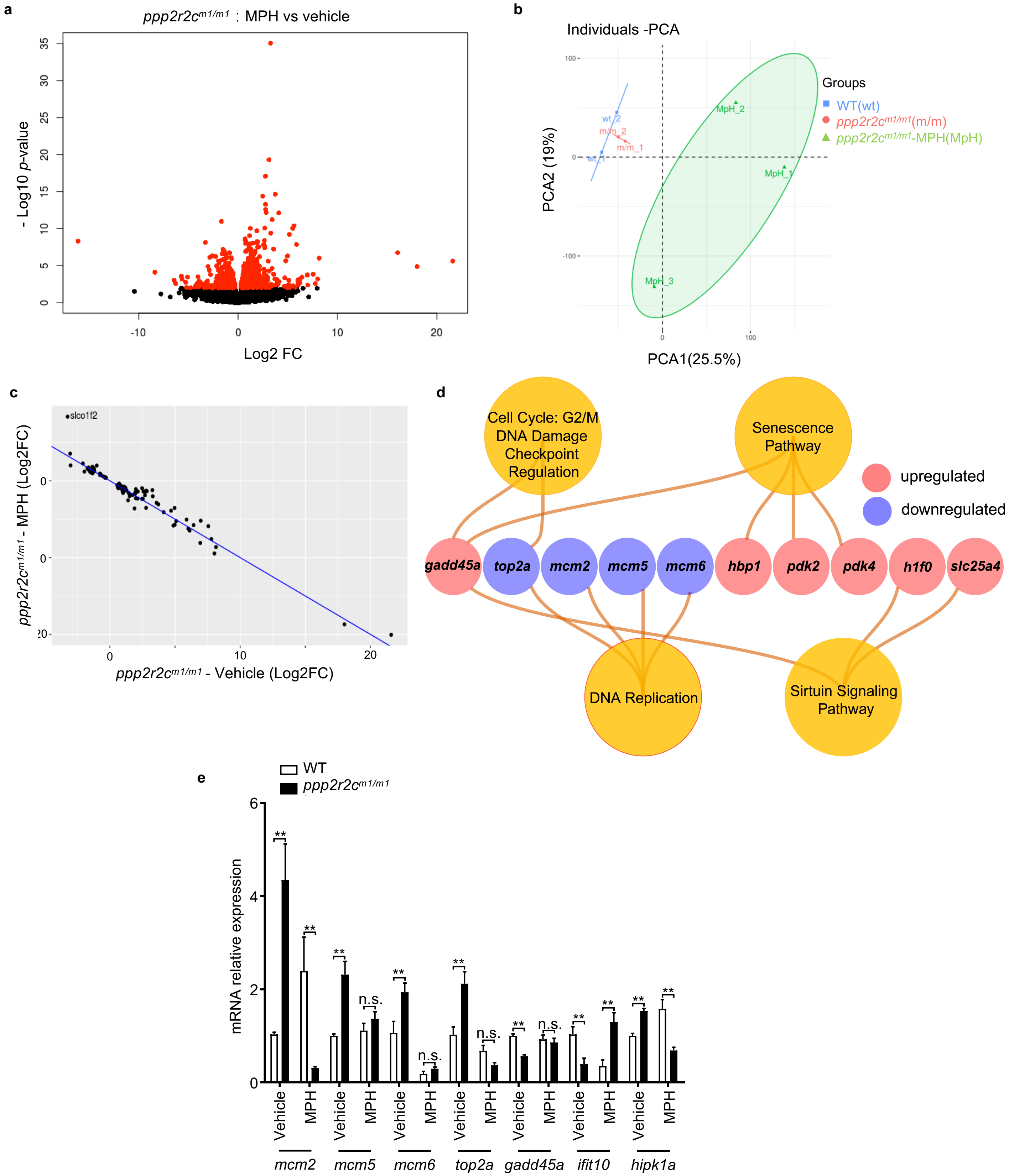

Supplementary Fig. 8

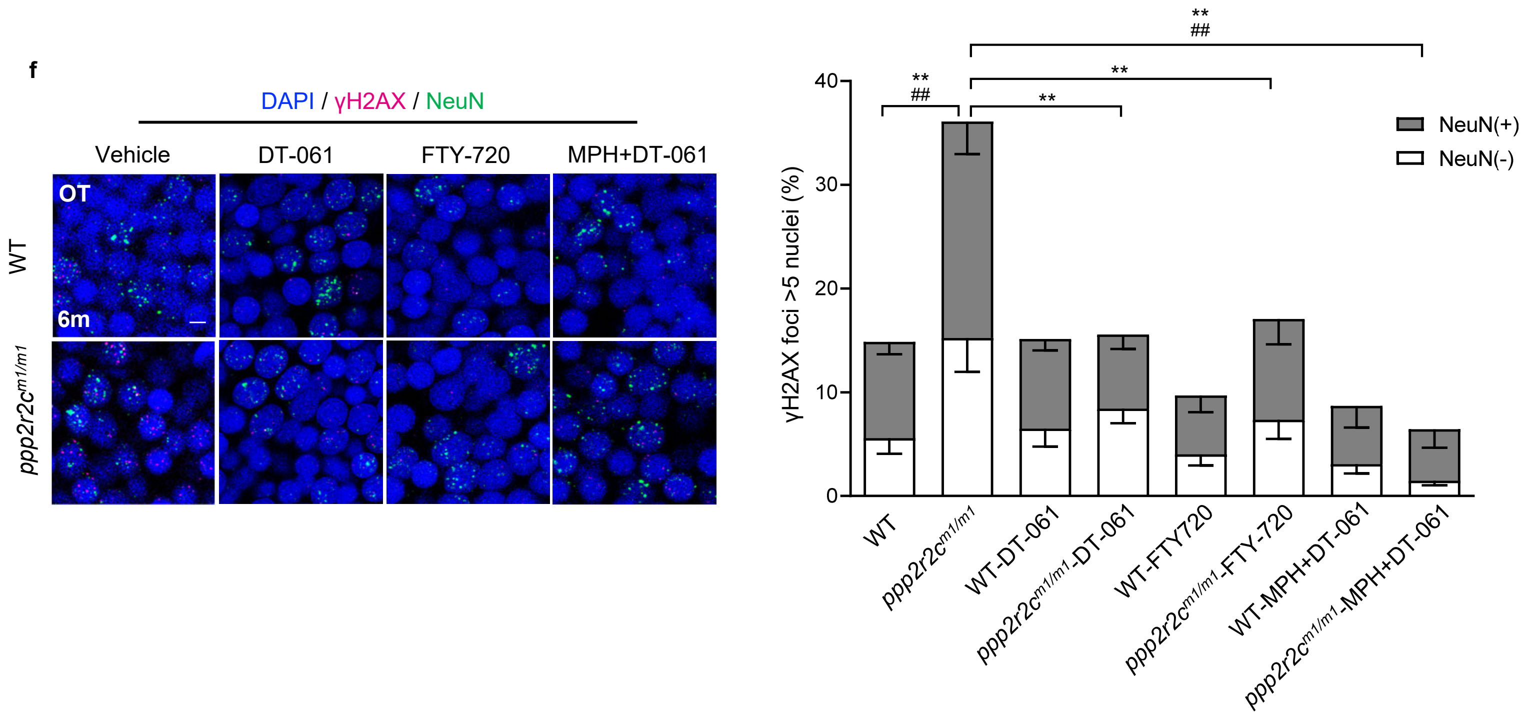

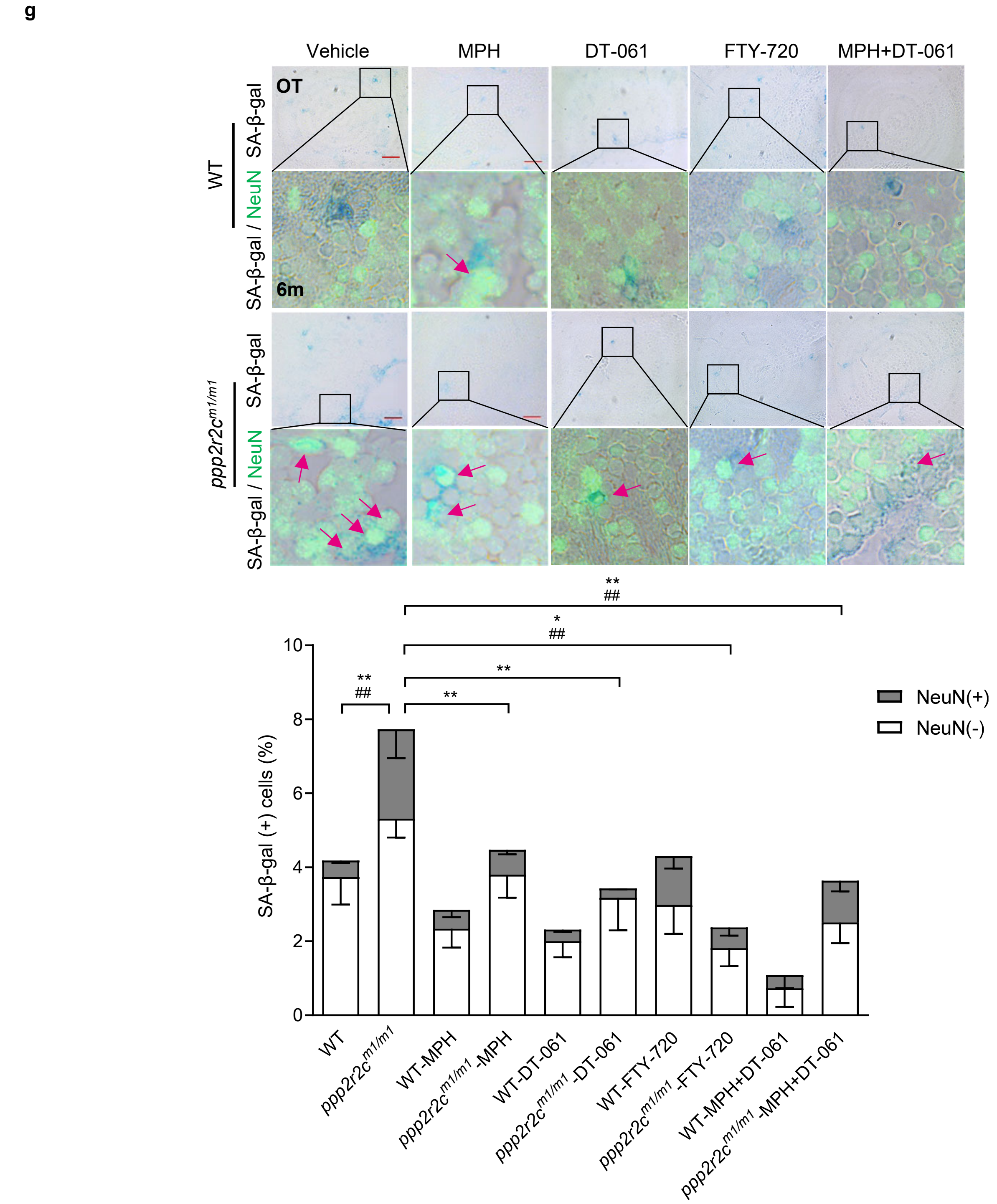

Supplementary Fig. 9

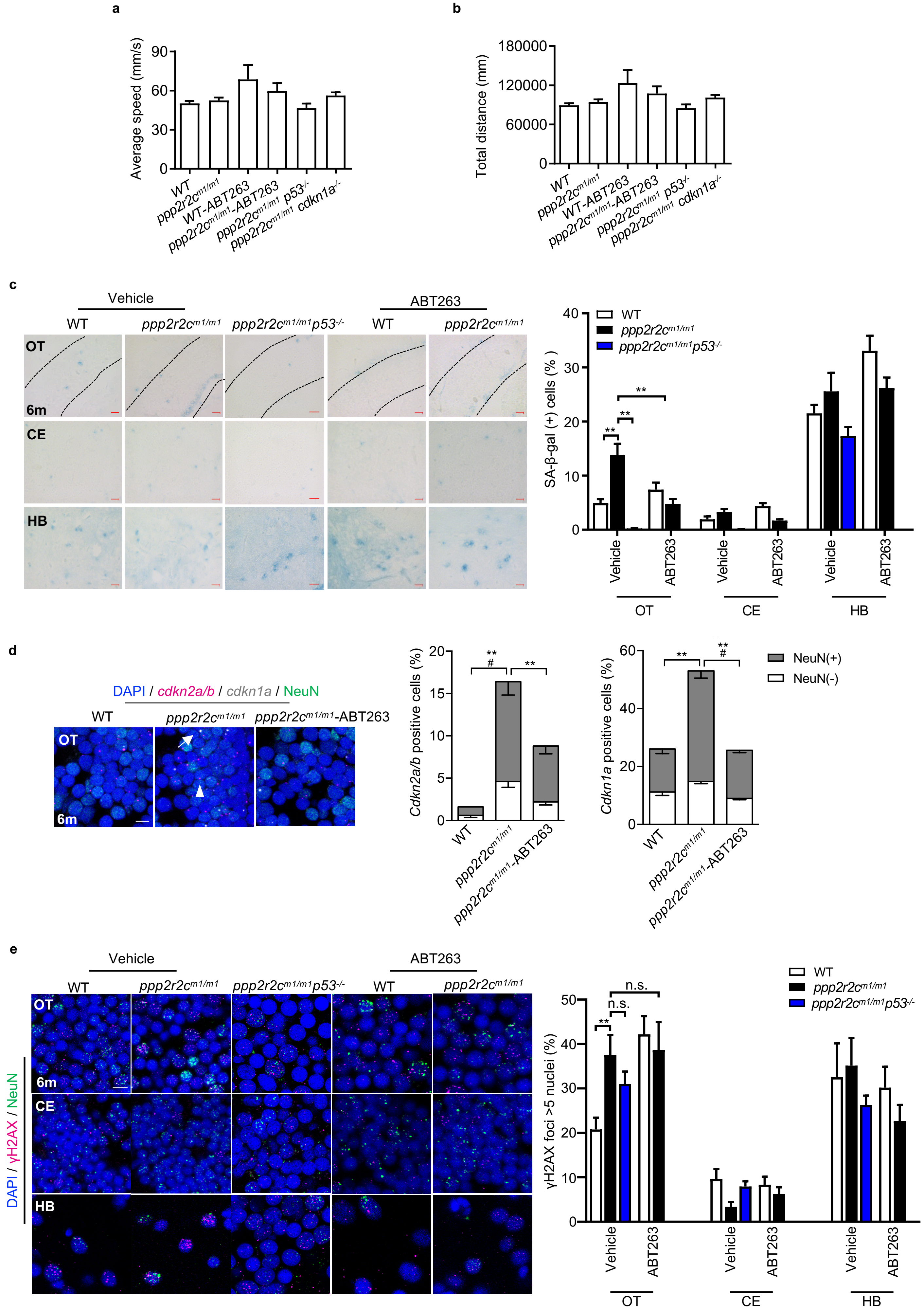

Supplementary Fig. 10

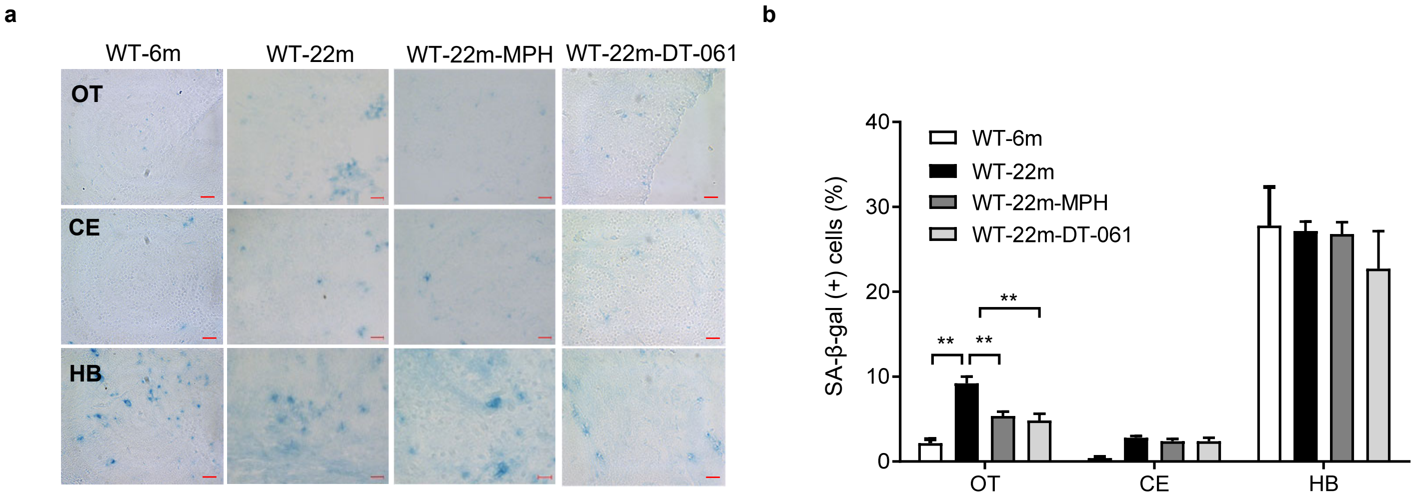

Supplement: Supplementary file 6 — Figures S1‐S10 [file ACEL-22-e13780-s006.pdf]
