# Supplementary material for: Protein phosphatase 2A activators reverse age‐related behavioral changes by targeting neural cell senescence
Source: Aging Cell. 2023 Jan 16;22(3):e13780. doi: 10.1111/acel.13780 (PMC10014060; doi:10.1111/acel.13780)
Supplement: Supplementary file 7 — Appendix S2 [file ACEL-22-e13780-s007.docx]

**SUPPLEMENTARY INFORMATION LEGEND**

**Supplementary Fig. 1. MPH ameliorates the behavioral disabilities of old mice**

**a** PP2A phosphatase assay of the frontal and temporal lobe of mice brains (*n* = 5 for 3m; *n* = 8 for 14m; *n* = 8 for 14m-MPH; unpaired two-sided *t* test). **b** Western blot of PP2Ac in the brain of WT, WT-22m, WT-22m-MPH, WT-22m-DT-061 zebrafish*.* (*n* = 3 independent biological samples). **c** Average speed of fish in the open field test (n = 12 for WT-6m, n = 11 for WT-22m and WT-22m-MPH, n=9 for WT-22m-DT-061 and WT-22m-FTY720). **d** Experimental design of behavioral test in 3-month-old and 14-month-old mice after MPH or vehicle treatment. **e** Number of transitions between the bright and the dark chambers from the Light/dark Transition test (*n* = 5 for 3m; *n* = 8 for 14m; *n* = 8 for 14m-MPH; one-way ANOVA). **f** The longest learning latency at 5 sequential training days in the Morris Water Maze test; **g** The frequency of mice crossing the platform at 6^th^ testing day in the latency Morris Water Maze test (*n* = 6 for 3m; *n* = 12 for 14m; *n* = 13 for 14m-MPH; one-way ANOVA, Statistic difference are shown as 3m vs 14m (red *), 3m vs 14m-MPH (blue *)). Data are means ± SEM. **P* < 0.05, ** *P* < 0.01, n.s. not significant.

**Supplementary Fig. 2. Construction and characterization of *ppp2r2c-*compropmised zebrafish**

**a** Relative expression of *ppp2r2c* in different tissue of 6-month-old WT zebrafish. (n = 3 independent biological samples; one-way ANOVA) **b** Schematic diagram showing the mutations of zebrafish *ppp2r2c* gene using the CRISPR-Cas9 system. The yellow and red boxes show the targeted exon and domain, respectively. The red dotted line shows the 4-nucleotide deletion in *ppp2r2c*. Black triangles show the locations of the PCR primers designed to amplify *ppp2r2c* mRNA, which were uses in (**c**). **c** RT-qPCR analysis of mRNA expression of *ppp2r2c* [*ppp2r2c-p1, ppp2r2c-p2* and *ppp2r2c-p3* indicates different locations of the primers at *ppp2r2c*, before, including and after mutated region shown in (**b**)] and other regulatory subunit genes of PP2A (*ppp2r2a, ppp2r2b, ppp2r2d, ppp2r5c*) in adult WT and homozygous *ppp2r2c-*mutant fish brains (*n* = 3 independent biological samples; unpaired two-sided *t* test). **d** PP2A phosphatase assay of adult zebrafish (6-month-old) brain, kidney, liver, muscle and gut (*n* = 3 independent biological samples, every sample contained two brains; unpaired two-sided *t* test). **e** Western blot of PP2Ac in the brain of WT, *ppp2r2c^m1/m1^* and *ppp2r2c^m2/m2^* (6-month-old) (*n* = 3 independent biological samples). **f** Survival curves of WT (*n* = 199), *ppp2r2c^m1/m1^* (*n* = 207) and *ppp2r2c^m2/m2^* (*n* = 130)*.* Data are means ± SEM. ** *P* < 0.01.

**Supplementary Fig. 3.** **Behavioral characterization of the *ppp2r2c^m1/m1^* and *ppp2r2c^m2/m2^* compromised fish.**

**a** Representative diagram of locomotion activity of *ppp2r2c^m2/m2^* adult fish during the 30-second light-on period. **b** Quantification of the highly active state duration during the 30-second light-on period (see Method) (*n* = 6 for every group). **c** Average speed of fish in the open field test (n = 8 for WT, n = 9 for *ppp2r2c^m1/m1^* and n=6 for *ppp2r2c^m2/m2^*). **d** Representative movement tracks (gray lines) in the mirror attack assay of WT and *ppp2r2c^m2/m2^* (6-months-old) during the 5-minute trial. Blue boxes show the position of the mirror. **e** Mirror attacking latency at indicated timepoint in 5-minute trail. **f** Quantification of the number of mirror attacks in the 5-minute intervals (*n* = 6 for every group). **g** Representative movement tracks (gray lines) of WT and *ppp2r2m2^m2/m2^* (6-months-old) during 30-minute trial in the open field test. The red square shows the central area of the tank. **h** Central area latency at indicated timepoint in 30-minute trail. **i** Cumulative time that the adult fish stayed within the central area (*n* = 6 for each group). **j** Total distance that fish swim in the open field test (n = 8 for WT, n = 9 for *ppp2r2c^m1/m1^* and n=6 for *ppp2r2c^m2/m2^*). **k** Experimental scheme for the social contact test. **l** Representative social events (red dots) in the social assay of WT and *ppp2r2c^m1/m1^* (6-months-old) (left). Quantification of social events frequency in 60 min duration (n= 12 per group, fish of same gender were used in single test) (right). **m** Representative diagram of locomotion activity of WT and *ppp2r2c^m1/m1^* (6-months-old) during the 12 hours night period. Quantification of sleep duration (n=3 per group). Data are means ± SEM. * *P* < 0.05, ** *P* < 0.01, n.s. not significant; unpaired two-sided *t* test.

**Supplementary Fig. 4. Different sorts of PP2A activators reverse the behavioral disabilities of *ppp2r2c*-compromised fish**

**a** PP2A phosphatase assay of WT and *ppp2r2c^m1/m1^* (6-month-old) brain treated with or without MPH, DT-061 and FTY720 (*n* = 3 independent biological samples). **b** Representative diagram of locomotion activity of WT and *ppp2r2c^m1/m1^* (6-month-old) with or without DT-061, FTY720 and MPH+DY-061 treatment during the 30-second lights-on period. **c** Quantification of the highly active state duration during the 30-second light-on period in (**b**) (*n* = 9 for WT-vehicle, WT-DT-061 *ppp2r2c^m1/m1^*- vehicle and *ppp2r2c^m1/m1^*-DT-061, *n* = 10 for WT-FTY-720 and *ppp2r2c^m1/m1^*- FTY-720, n=11 for WT-MPH+DT-061 and *ppp2r2c^m1/m1^*- MPH+DT-061). **d** Representative movement tracks (gray lines) in the mirror attack assay of WT and *ppp2r2c^m1/m1^* (6-months-old), with or without DT-061, FTY720 and MPH+DT-061 treatment for 3 days, during the 5-minute trial. Blue boxes show the position of the mirror. **e** Quantification of the number of mirror attacks in the 5-minute intervals in (**d**) (*n* = 9 for WT-vehicle, WT-DT-061 *ppp2r2c^m1/m1^*- vehicle and *ppp2r2c^m1/m1^*-DT-061, *n* = 10 for WT-FTY-720 and *ppp2r2c^m1/m1^*- FTY-720, n=11 for *ppp2r2c^m1/m1^*- MPH+DT-061, n=12 for WT-MPH+DT-061). **f** Representative movement tracks (gray lines) of WT and *ppp2r2c^m1/m1^* (6-months-old), with or without DT-061, FTY720 and MPH+DT-061 treatment for 3 days, during the 30-minute trial in the open field test. The red box shows the central area of the tank. **g** Cumulative time that the adult fish stayed within the central area in (**f**) (*n* = 9 for WT-DT-061, *ppp2r2c^m1/m1^*- vehicle and *ppp2r2c^m1/m1^*-DT-061, *n* = 10 for WT-vehicle, WT-FTY-720 and *ppp2r2c^m1/m1^*- FTY-720, n=11 for WT-MPH+DT-061 and *ppp2r2c^m1/m1^*- MPH+DT-061). Data are means ± SEM. **P* < 0.05, ** *P* < 0.01; two-way ANOVA.

**Supplementary Fig. 5.** **Neurotransmitters levels in *ppp2r2c-*compromised fish.**

**a** Volcano plot for neurotransmitter analysis of WT and *ppp2r2c^m1/m1^* (6-months-old) brains by LC-MS/MS. Red symbols indicate neurotransmitter with significant differential expression (5 replications, *n* = 60 per replication).

**Supplementary Fig. 6. Transcriptome of *ppp2r2c* -compromised zebrafish**

**a** Volcano plot of the RNA-seq results from *ppp2r2c^m1/m1^* versus WT brains at 6-months-old. Red symbols indicate significantly differentially expressed genes (DEGs) (FDR < 0.05 by likelihood ratio test, analyzed in DESeq2). **b** Validation of the RNA-seq results from brains of *ppp2r2c^m1/m1^* compared with WT (6-month-old) by RT-qPCR (*n* = 3 independent biological samples). **c** Pathway enrichment among the differentially expressed genes identified by RNA-seq in brain of *ppp2r2c^m1/m1^* compared with WT (6-month-old). **d** Reactive oxygen species (ROS) assay detected by DCFH-DA probe in adult WT and *ppp2r2c^m1/m1^* brains (cell number = 124 for WT and 81 for *ppp2r2c^m1/m1^*). Data are means ± SEM. ** *P* < 0.01, unpaired two-sided *t* test.

**Supplementary Fig. 7. Optic Tectum specific localization of neural cell alteration in *ppp2r2c* -compromised zebrafish**

**a** Diagram of an adult zebrafish brain. Dm (medial portion of the dorsal telencephalon), Vd (dorsal portion of the dorsal telencephalon), Tp (telencephalon), Th (thalamus), Hy (hypothalamus), Dp (diencephalon), OT (optic tectum), CE (cerebellum), HB (hindbrain). **b** Representative confocal image of NeuN (green) and γH2AX (magenta) staining in the CE, HB, Hy, Th, Dm and Vd of WT and *ppp2r2c^m1/m1^* (6-months-old) (scale bars, 5µm). **c** Quantification of percentage of γH2AX positive nuclei (≥5 γH2AX foci) in (**b**) (*n* = 6, over 100 cells (CE) or 30 cells (HB, Hy, Th, Dm and Vd) were analyzed per replication; unpaired two-sided *t* test). **d** Representative confocal images of NeuN (green) and TUNEL (magenta) co-staining in the OT, CE, and HB of WT and *ppp2r2c^m1/m1^* (6 months old) (scale bars, 5µm). Quantification of percentage of Tunel signals in neuronal (NeuN+) and non-neuronal (NeuN−) cells (*n* = 6, over 100 cells (OT and CE) or 30 cells (HB) were analyzed per replication; unpaired two-side t test). **e** Relative *ppp2r2c* expression in different brain region of adult zebrafish brain (6-month-old) (*n* = 3 independent biological replications, every replication pool two samples; one-way ANOVA). **f** Representative confocal image of NeuN (green) co-stain with *ppp2r2c* mRNA (magenta) in the OT, CE, HB, Hy, Th, Dm and Vd of WT (6-months-old) (scale bars, 5µm). Quantification of *ppp2r2c* mRNA signals in neuronal (NeuN+) and non-neuronal (NeuN−) cells (*n* = 5 for each group and over 100 cells (OT and CE) or 30 cells (HB, Hy, Th, Dm and Vd) were analyzed per replication; unpaired two-sided *t* test). **g** Representative images of SA-β-gal co-staining with NeuN immunofluorescence signals (green) in the OT of 6-month-old WT and *ppp2r2c^m1/m1^* (scale bars, 15µm). The arrows point to the SA-β-gal+ neural cells. Black boxes indicate the enlarged regions. Quantification of SA-β-gal positive neuronal (NeuN+) and non-neuronal (NeuN−) cells (n = 6 fish for each group and over 200 nuclei were analyzed per fish; * represents statistical difference in NeuN+ group, ^#^ represents statistical difference in NeuN- group; unpaired two-sided *t* test). **h** Representative images of SA-β-gal staining in CE, HB, Hy, Th, Dm and Vd of WT and *ppp2r2c^m1/m1^* (6-months-old) (scale bars, 15µm). **i** Quantification of percentage of SA-β-gal-positive cells in (**h**) (*n* = 6, over 100 cells were analyzed per replication; unpaired two-sided *t* test). **j** Relative mRNA expression levels of *cdkn1a*, *cdkn2a/b* and key SASP components determined by RT-qPCR in WT and *ppp2r2c^m1/m1^* brains (n = 3 independent biological samples, every sample pool two brains; unpaired two-sided *t* test). **k** Immunofluorescence detection of γH2AX (red) in heart and kidney of WT and *ppp2r2c^m1/m1^* (6-months-old) (scale bars, 5µm). **l** Quantification of percentage of γH2AX positive nuclei (≥5 γH2AX foci) in (**k**) (n=6, over 100 cells were analyzed per replication; unpaired two-sided *t* test). **m** Representative images of SA-β-gal staining in the heart and kidney of WT and *ppp2r2c^m1/m1^* fish at 6-months-old (scale bars, 50µm). **n** Quantification of the percentage of SA-βgal+ cells in (**n**) kidney (n=6, over 200 cells were analyzed per replication; unpaired two-sided *t* test).Data are means ± SEM. **P* < 0.05, ** *P* < 0.01, ^##^ *P* < 0.01, n.s., not significant.

**Supplementary Fig. 8. Brain transcriptome of MPH treated *ppp2r2c*-compromised fish and PP2A activators reduces the rate of neural cells with senescence markers in *ppp2r2c*-compromised fish.**

**a** Volcano plot of DEGs in 6-month-old fish of *ppp2r2c^m1/m1^*-MPH versus *ppp2r2c^m1/m1^*-vehicle. Red symbols indicate genes showing significant differential expression (FDR < 0.05 by likelihood ratio test, analyzed in DESeq2). **b** Principal component analysis (PCA) of RNA-seq data from WT (blue), *ppp2r2c^m1/m1^* (red) and *ppp2r2c^m1/m1^*-MPH (green) (*n* = 2 for WT and *ppp2r2c^m1/m1^*; *n* = 3 for *ppp2r2c^m1/m1^*-MPH). **c** Scatter plot of differentially expressed genes (DEGs) in the brain of *ppp2r2c^m1/m1^*-MPH vs. *ppp2r2c^m1/m1^*-vehicle (6-month-old). Black symbols indicate differentially expressed genes (n = 3 independent biological samples, every sample pooled two brains). **d** Ingenuity pathway analysis of shared genes indicated by the symbols in (**c**). Peripheral circles show enriched pathways (Fisher's exact test, FDR<0.05) and inner circles show the upregulated (red) and downregulated (blue) DEGs. **e** Validation of the differentially expressed genes detected by RNA-seq in the *ppp2r2c^m1/m1^*-MPH versus *ppp2r2c^m1/m1^*-vehicle groups at 6 months old by RT-qPCR (*n* = 3 independent biological samples; unpaired two-sided *t* test). **f** Immunofluorescence detection of NeuN (green) and γH2AX (magenta) in the OT of WT and *ppp2r2c^m1/m1^* (6-month-old) treated with or without DT-061, FTY720 and MPH+DT-061 for 3 days (scale bars, 5µm). Quantification shows the percentage of γH2AX positive (positive values indicate at least five γH2AX foci in the nucleus) neuronal (NeuN+) and non-neuronal (NeuN−) nuclei, (n = 6 per group, over 100 cells were analyzed per replication; * represents statistical difference in NeuN+ group, ^#^ represents statistical difference in NeuN- group; two-way ANOVA). **g** Representative images of SA-β-gal co-staining with NeuN (green) immunofluorescence in the OT of WT and *ppp2r2c^m1/m1^* (6-month-old) treated with or without MPH, DT-061, FTY720 and MPH+DT-061 for 3 days (scale bars, 15µm). Arrows point to the SA-β-gal+ neural cells. Quantification shows the percentage of neuronal SA-β-gal positive neuronal (NeuN+) and non-neuronal (NeuN−) cells (n = 6 per group, over 200 cells were analyzed per replication; * represents statistical difference in NeuN+ group, ^#^ represents statistical difference in NeuN- group; two-way ANOVA). Data are means ± SEM. * *P* < 0.05, ** *P* < 0.01, ^#^ *P* < 0.05, ^##^ *P* < 0.01, n.s., not significant.

**Supplementary Fig. 9. ABT263 treatment and *p53* knockout decrease the rate of senescent neural cells.**

**a** Average speed of fish in the open field test (n=10 for WT*-*vehicle*, ppp2r2c^m1/m1^-vehicle* *and ppp2r2c^m1/m1^cdkn1a^−/−^ groups;* n=9 for WT*-*ABT263*, ppp2r2c^m1/m1^-*ABT263 and *ppp2r2c^m1/m1^p53^−/−^*; one-way ANOVA). **b** Total distance that fish swim in the open field test (n=10 for WT*-*vehicle*, ppp2r2c^m1/m1^-vehicle* *and ppp2r2c^m1/m1^cdkn1a^−/−^ groups;* n=9 for WT-ABT263*, ppp2r2c^m1/m1^-*ABT263 and *ppp2r2c^m1/m1^p53^−/−^*; one-way ANOVA). **c** Representative images of SA-β-gal staining in WT-vehicle, *ppp2r2c^m1/m1^*-vehicle, WT-ABT263, *ppp2r2c^m1/m1^*-ABT263, and *ppp2r2c^m1/m1^p53^−/−^* brains at 6 months old (scale bars, 15µm) (left). Quantification of SA-β-gal-positive cells (right) (*n* = 4 for each group, over 200 cells were analyzed per replication; two-way ANOVA). **d** Representative images of confocal section of NeuN (green) co-staining with *cdkn2a/b* (magenta) or *cdkn1a* (grey) mRNA in the OT of WT, *ppp2r2c^m1/m1^* and *ppp2r2c^m1/m1^*-ABT263 at 6-month-old (scale bars, 5µm). Arrows and triangles point to the *cdkn2a/b and cdkn1a* mRNA signal respectively. Quantification of the percentage of *cdkn2a/b,* *cdkn1a* positive cells respectively (positive values indicate at least one mRNA signal) and percentage of double positive cells (*n* = 5 for each group and over 100 nuclei were analyzed per replication; * represents statistical difference in NeuN(+) group, *^#^* represents statistical difference in NeuN(-) group; one-way ANOVA). **e** Immunofluorescence detection of NeuN (green) and γH2AX (magenta) signals in WT-vehicle, *ppp2r2c^m1/m1^*-vehicle, WT-ABT263, *ppp2r2c^m1/m1^*-ABT263, and *ppp2r2c^m1/m1^p53^−/−^* brains at 6 months old (scale bars, 5µm) (left). Quantification of nuclei with at least five γH2AX foci (right) (*n* = 5 for each group, over 100 cells (OT and CE) or 30 cells (HB) were analyzed per replication; two-way ANOVA). Data are means ± SEM. * *P* < 0.05, ** *P* < 0.01, ^#^ *P* < 0.05, ^##^ *P* < 0.01, n.s. not significant.

**Supplementary Fig. 10. PP2A activators alleviates neural cells with senescence markers in old mice and fish.**

**a** Representative images of SA-β-gal staining in 22-month-old WT fish treated with or without MPH or DT-061 for 3 days (scale bars, 15µm). **b** Quantification of percentage of SA-β-gal positive cells shown in (**a**). (n=3 for 6m, n = 6 for 22m and 22m-MPH, n=4 for WT-DT-061 and over 200 nuclei were analyzed per fish; one-way ANOVA). Data are shown in means ± SEM. **P* < 0.05, ** *P* < 0.01.
